# Gut microbiota of miR‐30a‐5p‐deleted mice aggravate high‐fat diet‐induced hepatic steatosis by regulating arachidonic acid metabolic pathway

**DOI:** 10.1002/ctm2.70035

**Published:** 2024-10-03

**Authors:** Ruiying Wang, Xiaocheng Zhang, Yutian Wang, Yijun Lin, Yuling Zhou, Yan Wang, Gang Li

**Affiliations:** ^1^ Xiamen Cardiovascular Hospital of Xiamen University School of Medicine, Xiamen University Xiamen Fujian P. R. China; ^2^ Xiamen Key Laboratory of Cardiovascular Diseases Xiamen Cardiovascular Hospital of Xiamen University Xiamen Fujian P. R. China; ^3^ Department of Cardiology Nanfang Hospital Southern Medical University Guangzhou Guangdong P. R. China

**Keywords:** arachidonic acid metabolism, COX/LOX pathways, gut microbiota, hepatic steatosis, miR‐30a‐5p

## Abstract

**Background:**

Patients with non‐alcoholic fatty liver disease (NAFLD) often exhibit hepatic steatosis and dyslipidemia. Studies have shown that intestinal microorganisms are closely related to the occurrence of NAFLD and atherosclerosis. Our previous study has underscored the protective role of microRNA‐30a‐5p (miR‐30a‐5p) against atherosclerosis.

**Methods and Results:**

In the present study, we aimed to elucidate the effect and underlying mechanism of the intestinal microorganisms of miR‐30a‐5p knockout (KO) mice on NAFLD. Our findings demonstrated that KO exacerbated high‐fat diet (HFD)‐induced hepatic steatosis and disrupted liver function, as evidenced by elevated levels of total cholesterol, low‐density lipoprotein, alanine aminotransferase, aspartate transaminase, and total bile acids in serum. Fecal microbiota from HFD‐fed KO mice induced hepatic steatosis, dyslipidemia, and higher levels of enzymes indicative of liver damage in wild‐type mice. Remarkably, KO mice significantly intensified the above effects. 16s rDNA sequencing and metabolomics of the intestinal microbiota in the HFD‐treated KO and WT mice showed that the loss of miR‐30a‐5p resulted in intestinal microbiota imbalance and was highly related to the arachidonic acid metabolic pathway. Targeted metabolomic in the liver tissues unveiled upregulation of COX‐related (PGF2a, 8‐iso‐PGF2a and PGF2) and LOX‐related (LTB4, LTD4, 12S‐HETE and 15S‐HETE) factors in HFD‐treated KO mice. Immunohistochemistry and transcriptional analyses showed that miR‐30a‐5p affected arachidonic acid metabolism through the LOX/COX pathways. Besides, COX/LOX pathways and hepatic steatosis were reversed after reintroducing miR‐30a‐5p in HFD‐treated KO mice.

**Conclusions:**

This study reveals the pivotal mechanism by which miR‐30a‐5p and intestinal microbes regulate hepatic steatosis and abnormal lipid metabolism, offering promising avenues for NAFLD and atherosclerosis therapeutics.

**Highlights:**

MiR‐30a‐5p deletion aggravated hepatic steatosis and lipid disorder induced by an HFD in mice. Gut microbiota participated in the regulation of hepatic steatosis in the context of miR‐30a‐5p. Gut microbiota metabolism‐related arachidonic acid metabolic pathway contributed to miR‐30a‐5p‐regulated hepatic steatosis and lipid disorder. Reintroducing miR‐30a‐5p reversed hepatic steatosis and arachidonic acid metabolism disorder caused by HFD and miR‐30a‐5p deletion.

## Graphical abstract

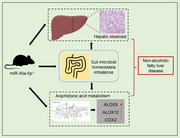

## 1 INTRODUCTION

With the lifestyle change and the prevalence of high‐calorie diets, non‐alcoholic fatty liver disease (NAFLD) has affected global health.^1^, ^2^ Notably, the main cause of death in NAFLD patients is cardiovascular disease.^3^ The pathogenesis of NAFLD is multifaceted, aligning with the ‘multiple‐hit model’, which implicates metabolic disorders, genetic susceptibility, epigenetics, and cell/organ interaction. Given the complexity of factors contributing to NAFLD, the development of therapeutic interventions remains challenging, necessitating a comprehensive approach.^4^ Interestingly, besides storing lipids, the adipose tissues in NAFLD patients also contains a large number of microRNAs (miRNAs), which act as a post‐transcriptional regulator and/or activate signal pathways to regulate the expression of target genes.^5^

Among miRNAs, miR‐30a‐5p plays a regulatory role in cardiovascular diseases such as heart failure, myocardial ischemia‐reperfusion injury, myocardial infarction and hypertension.^6^, ^7^, ^8^, ^9^ Besides, miR‐30a‐5p also has some research in tumour treatment. Wang et al found that miR‐30a‐5p in exosomes promoted angiogenesis and vascular permeability through PDCD10, thus promoting the development of intrahepatic cholangiocarcinoma, and targeting miR‐30a‐5p and apatinib played a synergistic anti‐cancer role.^10^ As for bacteriostasis, it is found that rESAT‐6 regulated calcium‐induced autophagy by regulating the expression of miR‐30a, while miR‐30a could kill intracellular mycobacteria by inhibiting anti‐autophagy reaction.^11^ MiRNAs, such as miR‐30a‐5p, are enriched in exosomes and participate in complex communication processes among cells or organs, such as the gut‐liver axis and heart‐brain axis.^12^ These information exchanges are essential to the homeostasis of the body, with any disruption potentially precipitating disease pathology.

The bidirectional relationship between the liver and gut microbiota is called the ‘gut‐liver axis’. Gut microbiota or their metabolites enter the portal vein through the damaged intestine to further cause hepatitis, and the liver responds to intestinal cues by secreting bile acids and other active substances.^1^, ^4^, ^13^ The diet containing probiotics (*Lactobacillus lactis* and *Pediococcus pentosaceus*) could improve the metabolites of short‐chain fatty acids, bile acids and tryptophan by regulating gut microbiota and anti‐inflammatory metabolites, thus inhibiting the process of NAFLD.^14^ So, the gut microbiota and its metabolites were closely related to the homeostasis of the host body, and the changes of these components could provide ideas for the treatment and diagnosis of liver‐related diseases. In our previous study, we found that miR‐30a‐5p alleviated atherosclerosis and reduced serum lipids by modulating M1/M2 macrophage polarisation and lipid metabolism.^15^ However, whether miR‐30a‐5p can regulate hepatic steatosis or atherosclerosis through the gut microbiota remains uncertain. In this study, we found that miR‐30a‐5p played a regulatory role in hepatic steatosis and blood lipid metabolism mediated by the gut microbiota. Additionally, we further clarified the specific involvement of arachidonic acid metabolic pathway in regulating hepatic steatosis and blood lipid metabolism via the gut‐liver axis.

## 2 METHODS AND REAGENTS

### 2.1 Animals

C57BL/6J mice (6–8 weeks old, male) were purchased from GemPharmatech (Nanjing, China). We used CRISPR/Cas9 technology to construct miR‐30a‐5p^−/−^ mice with C57BL/6J genetic background, whose genotypes were verified by PCR and using the following primers: 5′‐AGCTTCCCTACTTTGGTGTTT‐3′; 5′‐ TGGTGTGTGTGAATTGACCT‐3′. All mice were kept in the specific pathogen‐free laboratory of the Animal Experimental Center of Xiamen University (22°C ± 2°C, humidity 50−70%, 12 h light/dark cycle). All animal studies were conducted in compliance with the guidelines of the Institutional Animal Care and Use Committee of Xiamen University and were approved by the Laboratory Animal Management and Ethics Committee of Xiamen University (Approval NO. XMULAC20190120).

### 2.2 Animal groups and treatment

For the treatment with a high‐fat diet (HFD, PAD, Dyets Biotechnology (Wuxi), Jiangsu, China), C57BL/6J wild‐type and miR‐30a‐5p^−/–^ mice were randomly assigned to two groups: normal diet (WT group, KO group) and high‐fat diet (WT + HFD group, KO + HFD group) for a duration of 8 weeks. Liver, blood and heart tissues were collected after sacrifice by inhalation of carbon dioxide.

To assess the impact of fecal microbiota transplantation from HFD‐fed miR‐30a‐5p^−/−^ mice on the liver and lipid metabolism of recipient mice, WT and miR‐30a‐5p^−/–^ mice (6‐8 weeks old, male) were subjected to HFD for 8 weeks, with fecal samples collected accordingly. Fecal microbiota were collected every two days and pooled within each group in sterile PBS (150 mg feces/1 mL PBS) and centrifuged at 500 rpm/min for 5 min. Before transplantation, recipient mice were treated with the antibiotic mixture (ABX), comprising vancomycin (.5 g/L, Cat#: 1161, BioFroxx, Germany), cefixime (1 g/L, Cat#: HY‐B1381, MedChemExpress, Shanghai, China) and metronidazole (1 g/L, Cat#: HY‐B0318, MedChemExpress, Shanghai, China) in drinking water for 2 weeks. Subsequently, the fecal microbiota were intragastric administrated to the recipient mice (6‐8 weeks old, male) for 2 weeks. Following transplantation, mice were maintained on a normal diet for an additional 8 weeks, as depicted in Figure 2A. Liver, blood and heart tissues were collected for further analysis upon sacrifice by inhalation of carbon dioxide.

To further verify the regulation of miR‐30a‐5p on the liver function and arachidonic acid metabolism, we injected 100 µL/each HBAAV2/9‐cTNT‐LUC (AAV‐NC) or HBAAV2/9‐cTNT‐mmu‐mir‐30a‐LUC (AAV‐OE, HH20230815FJCJY, HANBIO, Shanghai, China) into miR‐30a‐5p^−/−^ mice through the tail vein. Two weeks after injection, the mice began HFD for 8 weeks. Liver tissues and blood were collected after sacrifice by inhalation of carbon dioxide.

### 2.3 Histological analysis

Following collection, mouse liver samples were embedded in an optimal cutting temperature (OCT) compound (Cat#: G6059, Servicebio, Wuhan, China) and frozen at −80°C. Frozen sections of 6 µm thickness were obtained. For H&E staining, we used a hematoxylin‐eosin kit (Cat#: G1076, Servicebio, Wuhan, China) as the protocol of the manufacturer. Masson trichrome staining was carried out using a Masson staining solution (Cat#: G1006, Servicebio, Wuhan, China). Oil Red O staining was performed using an Oil Red O saturated solution (Cat#: G1015, Servicebio, Wuhan, China) for lipid staining. Photographs were captured using a Leica microscope.

### 2.4 Serum indicator detection

After treatment, mouse blood samples were collected and centrifuged at 3000 rpm for 10 min to obtain serum. Total cholesterol (Cat#: AUZ0167, Beckman Coulter, Brea, CA, USA), triglyceride (Cat#: AUZ0161, Beckman Coulter, Brea, CA, USA), high‐density lipoprotein‐cholesterol (HDL‐c, Cat#: AUZ0195, Beckman Coulter, Brea, CA, USA), low‐density lipoprotein‐cholesterol (LDL‐c, Cat#: AUZ0386, Beckman Coulter, Brea, CA, USA), alanine aminotransferase (ALT, Cat#: AUZ0528, Beckman Coulter, Brea, CA, USA), aspartate aminotransferase (AST, Cat#: AUZ0263, Beckman Coulter, Brea, CA, USA), cholinesterase (Cat#: AUZ0163, Beckman Coulter, Brea, CA, USA) and total bile acids (Cat#: AUZ0266, Beckman Coulter, Brea, CA, USA) levels in mouse serum were quantified using the Beckman Coulter AU biochemical analysis system (Beckman Coulter, CA, USA).

### 2.5 16S rDNA sequencing

The results of 16S rDNA amplicon sequencing were performed and analysed by Applied Protein Technology Co., Ltd (Order NO: P20220502318, Shanghai, China). The genomic DNA of the gut contents from WT + HFD mice (*n* = 7) and KO + HFD mice (*n* = 7) was extracted using the cetyltrimethylammonium bromide (CTAB) and sodium dodecyl sulfate (SDS) method. DNA purity and concentration were assessed, and the selected V3‐V4 variable regions were amplified by PCR using specific primers with barcodes and high‐fidelity DNA polymerase: 16S V3‐V4 (341F: CCTAYGGGRBGCASCAG; 806R: GGACTACNNGGGTATCTAAT). PCR products were separated using 2% agarose gel electrophoresis, and the target fragments were excised. An AxyPrepDNA gel recovery kit (AP‐GX‐250, AXYGEN, CA, USA) was used for gel recovery. According to the preliminary quantitative results of electrophoresis, the recovered products of PCR amplification were detected and quantified by QuantiFluor™‐ST blue fluorescence quantitative system (Promega, Wisconsin, USA), and mixed according to the requirements of sequencing quantity of each sample. NEB Next ®Ultra library DNA Library Prep kit (E7645, NEB, USA) was employed for library construction, with library quality assessed using Agilent Bioanalyzer 2100 and Qubit. Upon passing the quality test, libraries were sequenced, followed by Operational Taxonomic Units (OTU) clustering and species classification analysis based on valid data. Based on the results of the OTU cluster analysis, we analysed the diversity index of OTU and detected the sequencing depth. Based on taxonomic information, statistical analysis of community structure was carried out at various taxonomic levels.

### 2.6 Metabolomics

Untargeted metabolomics detection and analysis were conducted by Applied Protein Technology Co., Ltd (Order NO: P20221006501, Shanghai, China). Gut contents from WT + HFD mice (*n* = 8) and KO + HFD mice (*n* = 8) were separated by an ultra‐performance liquid chromatography system (Agilent 1290 Infinity LC, CA, USA), detected using a mass spectrometer (AB Sciex Triple TOF 6600, CA, USA), and then analysed in both electrospray ionisation positive and negative ion modes. The XCMS analysis software (xcmsonline.scripps.edu) was used for analysis. The variable importance (VIP) value of each variable in the OPLS‐DA model in projection was calculated, and the significance of differences between the two independent samples was determined by the *t*‐test. Metabolites exhibiting significant changes were identified based on criteria of VIP > 1 and *p* < .05.

### 2.7 Targeted metabolomics

Targeted metabolomics detection and analysis were performed by Applied Protein Technology Co., Ltd (Order NO: YAS202306290056, Shanghai, China). Liver tissues from WT + HFD mice (*n* = 9) and KO + HFD mice (*n* = 9) were separated using an ultra‐performance liquid chromatography system (Agilent 1290 Infinity LC, CA, USA) and detected using a mass spectrometer (5500 QTRAP, SCIEX, CA, USA). Chromatographic peak areas and retention times were extracted using the Multiquant 3.0.2 software. Retention times were adjusted using standard substance correction for the target compounds, and the metabolites were identified.

### 2.8 Real‐time PCR

After treatment, the livers of mice were collected. The total RNA of the liver tissues was then isolated using the TRIzol/chloroform/isopropanol method. The total RNA was reverse transcribed into cDNA using a reverse transcription kit (Cat#: RR047A, Takara Biomedical Technology, Beijing, China). RNA levels were quantified using a quantitative PCR kit (Cat#: 11204ES08, Yeasen, Shanghai, China) with the following primer sets: ALOX5 (F:5′‐AGGCGAGGGCGAGATTGTC‐3′; R: 5′‐ CGTCTGTGCTGCTTGAGGATG‐3′), ALOX12 (F:5′‐CAGGTAGTGAGCACAGGTGGAG‐3′; R: 5′‐GCCAGATCATCAGGAGGACAGAG‐3′), ALOX15 (F:5′‐AGTTCCTCACCTACTCTACACCATG‐3′; R: 5′‐CCTCCTCCACCTGTGCTCATC‐3′), COX1 (F:5′‐CCAGAACCAGGGTGTCTGTGTC‐3′; R: 5′‐ACAGTTGGGGCCTGAGTAGC‐3′), and COX2 (F:5′‐ACCCATCAGTTTTTCAAGACAGAT‐3′; R: 5′‐GCGCAGTTTATGTTGTCTGT‐3′).

### 2.9 Immunohistochemistry

After treatment, mouse liver samples were embedded in the OCT agent (Cat#: G6059, Servicebio, Wuhan, China) and frozen at −80°C. Frozen sections of 6 µm thickness were obtained using the freezing microtome (Leica 1950, Germany). Sections were incubated with primary antibodies against ALOX5 (Cat#: A2158, Abclonal, Wuhan, China), ALOX12 (Cat#: A14703, Abclonal, Wuhan, China), ALOX15 (Cat#: A6864, Abclonal, Wuhan, China), COX1 (Cat#: A7341, Abclonal, Wuhan, China), COX2 (Cat#: A3560, Abclonal, Wuhan, China). The sections were then incubated with the corresponding secondary antibody (HRP labelled goat anti‐rabbit IgG, Cat#: GB23303, Servicebio, Wuhan, China) for 1 h. Staining was visualised using a DAB solution (Cat#: SW1020, Solarbio, Beijing, China) and counterstained with hematoxylin. The images were obtained using a microscope (Leica, Germany). Positive staining appeared as brownish yellow, while nuclei stained with hematoxylin appeared blue. The average optical density (AOD) was measured and analysed by Image J (IHC toolbox plugin) software.

### 2.10 Statistical analysis

Data are expressed as means ± standard deviations (Mean ± SD) and analysed using GraphPad Prism 9. Normally distributed data (2 groups) were analysed by Student's *t*‐test. One‐way analysis of variance (ANOVA) followed by either the Tukey test or Dunnett test was employed for multiple comparisons (> 2 groups) as deemed appropriate. Statistical significance was defined as a two‐sided *p *< .05.

## 3 RESULTS

### 3.1 Deletion of miR‐30a‐5p alters liver structure and function after HFD treatment

**FIGURE 1:**
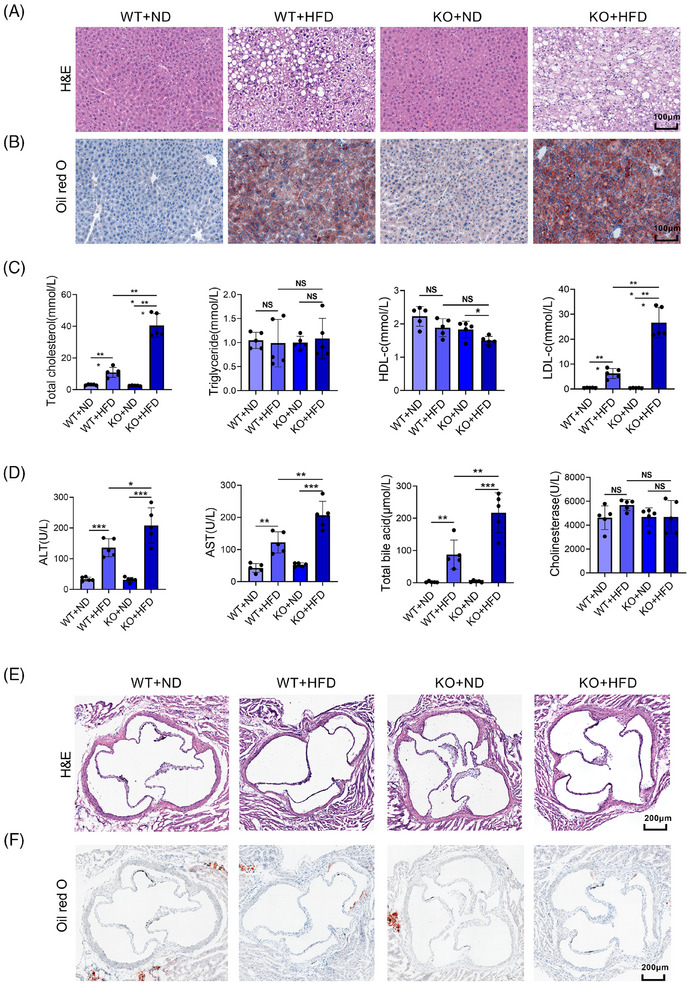
The deletion of miR‐30a‐5p aggravated liver damage and the increase of blood lipids after HFD. (A) Representative images of H&E staining in the liver of WT/KO mice treated with normal diet (ND) or (high‐fat diet) HFD (*n* = 5, scale bar: 100 µm). (B) Representative images of Oil Red O staining in the liver of WT/KO mice treated with ND or HFD (*n* = 5, scale bar: 100 µm). (C) Total cholesterol, triglyceride, HDL‐c, and LDL‐c levels in the serum of WT/KO mice treated with ND or HFD (*n* = 5). (D) Serum indicators of liver function including ALT, AST, total bile acid, and cholinesterase were detected in the WT/KO mice treated with ND or HFD (*n* = 5). (E) Representative images of H&E staining in the aortic root of WT/KO mice treated with ND or HFD (*n* = 5, scale bar: 200 µm). (F) Representative images of Oil Red O staining in the aortic root of WT/KO mice treatment with ND or HFD (*n* = 5, scale bar: 200 µm). Data are expressed as mean ± SD and one‐way ANOVA with Tukey's test was used for multiple comparisons, NS indicated no significant difference, ^*^ *p *< .05, ^**^ *p *< .01, ^***^ *p *< .001.

To investigate the effect of miR‐30a‐5p deletion on liver function and lipid metabolism, we fed the WT and miR‐30a‐5p knockout (KO) mice with a high‐fat diet (HFD) or normal diet for 2 months and tested the liver histology, serum lipid levels, liver functions, and aortic root plaque formation. The deletion of miR‐30a‐5p in KO mice was confirmed by PCR and qPCR, as shown in Figure S1A and B. After treatment, there was increased liver adipose in WT mice after HFD treatment (Figure 1A). Conversely, HFD‐treated KO mice displayed augmented adipocyte size and quantity, accompanied by heightened inflammatory cell infiltration compared to HFD‐treated WT mice (Figure 1A). Consistent with H&E staining, Oil Red O staining revealed intensified red and dark colour in HFD‐treated KO mice compared to HFD‐treated WT mice (Figure 1B). We further evaluated blood lipid metabolism, including total cholesterol, triglyceride, high‐density lipoprotein‐cholesterol (HDL‐c), and low‐density lipoprotein‐cholesterol (LDL‐c). Results indicated that HFD treatment in WT mice led to elevated levels of total cholesterol and LDL‐c, which were further elevated in the HFD‐treated KO mice. The level of triglyceride had no significant change among the four groups, while the HDL‐C level demonstrated a sole reduction in the KO + HFD group (Figure 1C). Alanine aminotransferase (ALT) and aspartate aminotransferase (AST) are the key indicators for liver damage. Serum levels of ALT and AST in both WT and KO mice increased significantly after HFD treatment, particularly evident in the KO + HFD group (Figure 1D). The change in total bile acids was consistent with ALT and AST, but the level of cholinesterase remained largely unchanged across the four groups (Figure 1D). In a previous study,^15^ we have proved that the plaque in the aortic root of ApoE^−/−^ mice intervened by miR‐30a‐5p knockout adenovirus increased significantly compared with that of ApoE^−/−^ mice after high‐fat feeding. Thus, we assessed the aortic plaque formation of the above‐treated mice (Figure 1E and F). However, no significant differences in plaque formation were observed, potentially attributed to the lower sensitivity of C57BL/6J mice to plaque development. In brief, the deletion of miR‐30a‐5p in mice exacerbates hepatic injury and steatosis, along with elevated blood lipid levels following HFD.

### 3.2 Gut microbiota is the key factor influencing liver damage in KO mice

**FIGURE 2:**
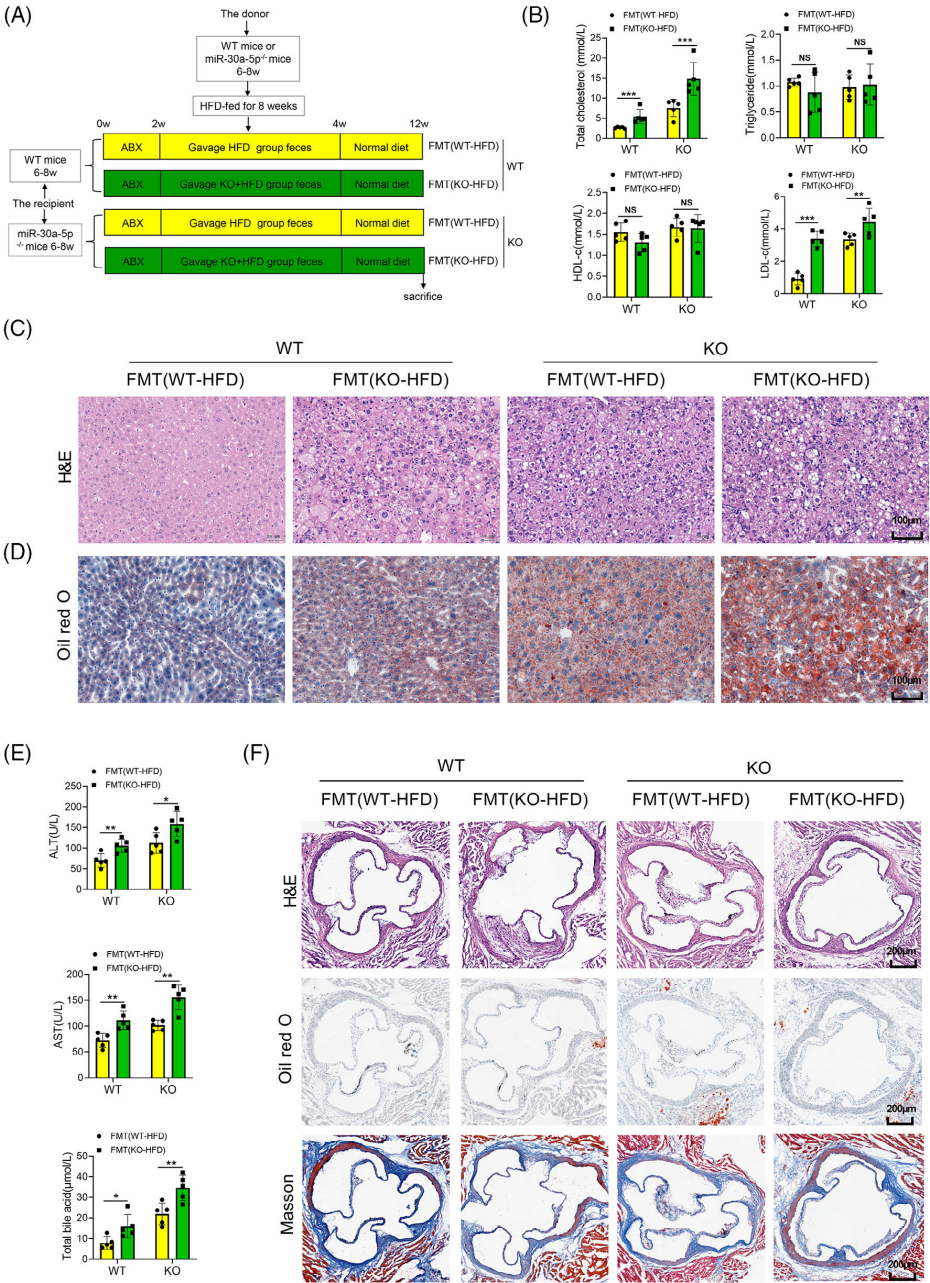
Transplantation with fecal microorganisms of the HFD‐treated KO mice aggravated the liver function and structure in WT and KO mice. (A) The schematic of the experimental timeline and design of the WT and ApoE^−/−^ mice treatment. WT or KO mice (6–8 weeks old, male) were treated with ABX for 2 weeks, transplanted with intestinal microorganisms (FMT) of WT or KO mice treated with HFD, and then fed a normal diet for 8 weeks. (B) Total cholesterol, triglyceride, HDL‐c, and LDL‐c levels in the serum of WT or KO mice treatment with FMT (WT‐HFD) or FMT (KO‐HFD) (*n* = 5). (C) Representative images of H&E staining of liver tissues in WT or KO mice treatment with FMT (WT‐HFD) or FMT (KO‐HFD) (*n* = 5, scale bar: 100 µm). (D) Representative images of Oil Red O staining of liver tissues in WT or KO mice treatment with FMT (WT‐HFD) or FMT (KO‐HFD) (*n* = 5, scale bar: 100 µm). (E) The levels of ALT, AST, and total bile acid in the serum of WT or KO mice treated with FMT (WT‐HFD) or FMT (KO‐HFD) (*n* = 5). (F) Representative images of H&E staining, Oil Red O, and Masson staining of the aortic roots in WT or KO mice treatment with FMT (WT‐HFD) or FMT (KO‐HFD) (*n* = 5, scale bar: 200 µm). Data are expressed as mean ± SD and one‐way ANOVA with Tukey's test was used for multiple comparisons, NS showed no significant difference, ^*^ *p *< .05, ^**^ *p *< .01, ^***^ *p *< .001.

To further ascertain the regulatory effect of intestinal microorganisms of KO mice on hepatic metabolism, after ABX treatment for 2 weeks, we transplanted fecal microorganisms from HFD‐treated WT or KO mice to the recipient WT or KO mice. Following transplantation for 2 weeks, a normal diet was fed to the mice for an additional 2 months (Figure 2A). The results showed that the levels of total cholesterol and LDL‐c in the serum of mice receiving FMT from the KO + HFD group were significantly higher compared to those receiving FMT from the WT + HFD group. The levels of total cholesterol and LDL‐c in the KO mice were consistently higher than those in the WT mice under the same treatment conditions (Figure 2B). However, there were no significant differences in the levels of triglyceride and HDL‐c in serum between the two groups, both in WT and KO mice (Figure 2B). Histological examination of liver tissue from mice receiving FMT from the KO + HFD group revealed increased liver adipose, inflammatory cells, and a looser arrangement of hepatocytes compared to the WT + HFD group, observed in both WT mice and in KO mice (Figure 2C and D). In addition, the indicators of liver function aligned with these observations that intestinal microbes of the KO + HFD group promoted levels of ALT, AST and total bile acids in the serum of both WT and KO mice (Figure 2E). These results indicated that irrespective of mouse genotype, liver structural and functional changes following transplantation of intestinal microorganisms from the KO + HFD group are more pronounced than those from the HFD group, with KO mice exhibiting a more significant response to these alterations. The results of H&E, Oil Red O, and Masson staining revealed no significant changes and no plaque in the aortic root of mice in each group (Figure 2F), consistent with the findings in Figure 1E and F. Therefore, the intestinal microorganisms of WT and KO mice exhibit differentiation after HFD treatment, and the phenotypic disparity in hepatic metabolism between WT and KO mice may be related to the heterogenisation of intestinal microorganisms.

To investigate the role of fecal microbiota of HFD‐treated ApoE^−/−^ mice on liver function and atherosclerosis, fecal microbiota from both ND‐ and HFD‐treated ApoE^−/−^ mice were collected and transplanted fecal microbiota to ApoE^−/−^ mice for 2 weeks (Figure S2). The result showed that plaques appeared in the aorta of ApoE^−/−^ mice after transplanting intestinal microorganisms of the HFD‐treated mice ApoE^−/−^ mice [FMT (ApoE^−/−^ + HFD)], but no plaques were observed after transplanting intestinal microorganisms in the ND‐treated mice [FMT (ApoE^−/−^ + ND)], as shown in Figure S2B and C. Consistent with these findings, histopathological examination in the aortic roots and liver using H&E, Oil Red O, and Masson staining supported plaque induction by FMT (ApoE^−/−^ + HFD) in ApoE^−/−^ mice (Figure S2). The levels of total cholesterol, triglyceride, LDL‐c, and HDL‐c were also increased after the FMT (ApoE^−/−^ + HFD) treatment (Figure S2H). These results demonstrate the dependency of plaque formation and hepatic metabolic function in ApoE^−/−^ mice on the activities of intestinal microorganisms.

### 3.3 Arachidonic acid metabolism of gut microorganisms is involved in the regulation of hepatic metabolism

**FIGURE 3:**
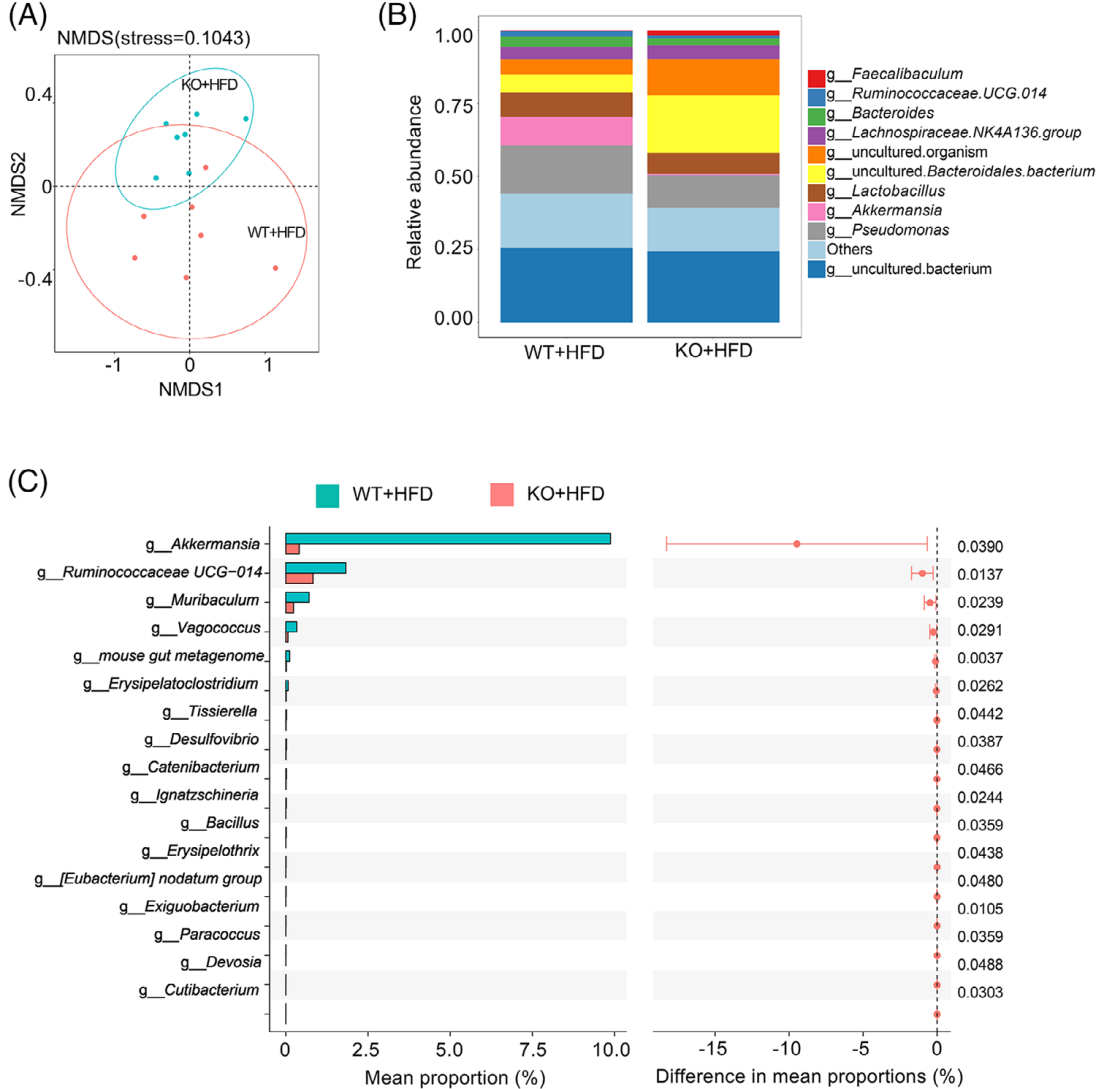
The differences in intestinal microorganisms between the HFD‐treated WT and KO mice. (A) Nonmetric multidimensional scaling (NMDS) diagram of the HFD‐treated WT (*n* = 7) and KO (*n* = 7) mice, where samples with similar community structures are clustered together. (B) The relative abundance histogram of the top 10 in the WT + HFD and KO + HFD groups at the genus level. (C) LDA Effect Size analysis showed the species that differ significantly between the WT + HFD and KO + HFD groups.

Based on the preceding results, we hypothesised that the intestinal microorganisms of miR‐30a‐5p KO may influence liver function. To investigate this, we applied 16S rDNA sequencing to examine the microbial composition differences between WT and KO mice after HFD induction. Analysis of the intestinal microbiota of the HFD‐treated WT and KO mice revealed substantial disparities in species composition and distribution between the two groups (Figure 3A). LDA Effect Size analysis further delineated distinct species of fecal microbiota between the two groups (Figure S3). Notably, the top 10 abundant species exhibiting significant differences between these groups comprised s_*Bacteroides.dorei*, s_*Escherichia.coli*, s_*Lactobacillus.reuteri*, s_*Bacteroides.fragilis*, s_uncultured.*Bacterioides.sp*, s_*Lactobacillus.murinus*, s_*Lactobacillus.gasseri*, s_uncultured.organism, s_uncultured.*Bacteroidales.bacterium*, s_uncultured.*Bacterium* and others (Figure 3B). Further analysis at the genus level revealed *g_Akkermansia* as the most significantly altered taxon between the WT + HFD and the KO + HFD groups (Figure 3C). Additionally, Kyoto Encyclopedia of Genes and Genomes (KEGG) and Clusters of Orthologous Groups of proteins (COG) analyses for function prediction indicated a significant impact on energy metabolism and energy production and conversion orchestrated by KO (Figure S4A and B). Studies have shown that *Akkermansia* can help mitigate NAFLD, obesity and cardiovascular diseases by regulating lipid metabolism and reducing inflammation.^16^ As such, *Akkermansia* has been identified as a promising ‘next‐generation beneficial microorganism’ for its health‐promoting properties.^17^ Our current findings, in conjunction with earlier research, suggest that the proportion of *Akkermansia* may be a significant factor for KO‐regulated liver dysfunction.

**FIGURE 4:**
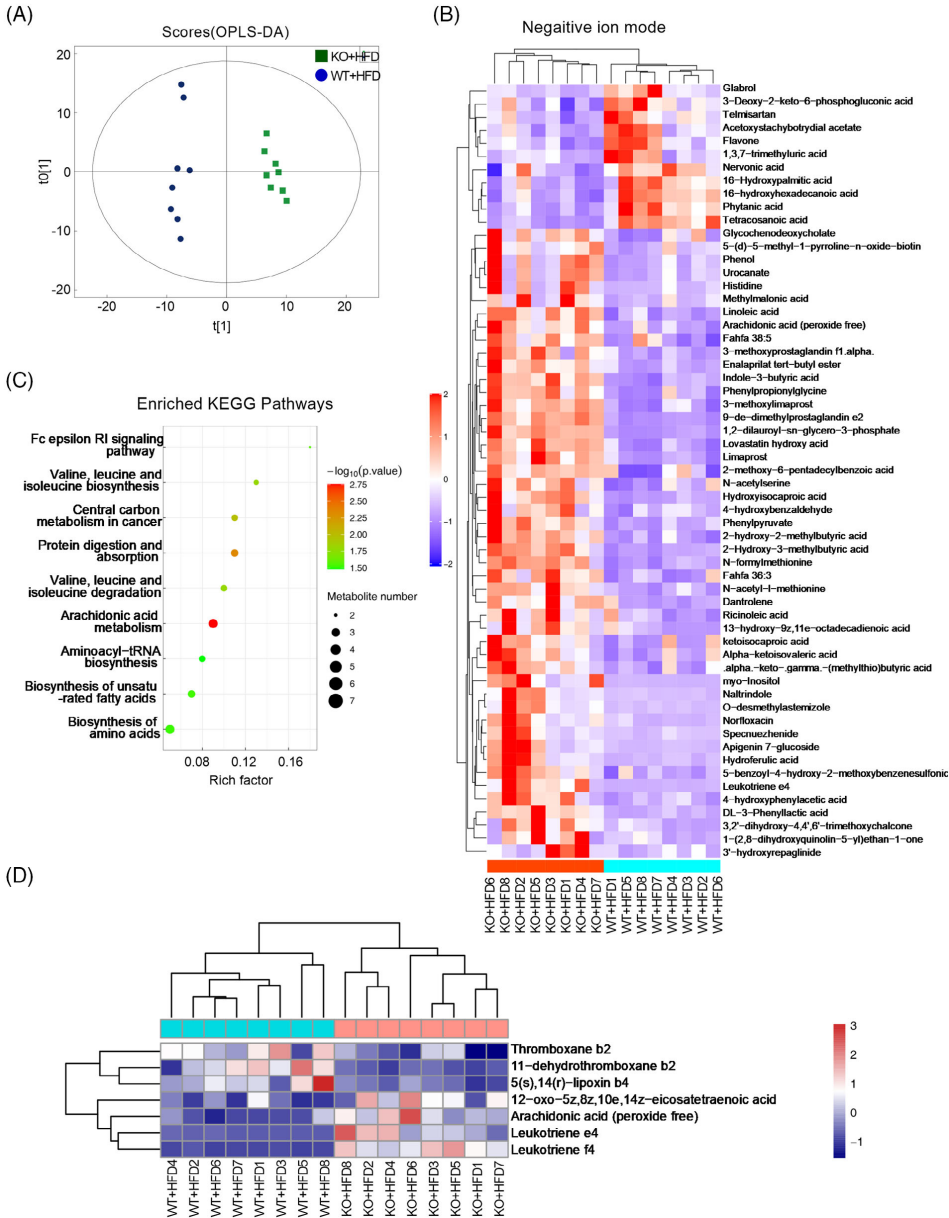
Metabolomics of gut microorganisms in the HFD‐treated WT and KO mice. (A) Partial least square discrimination analysis (PLS‐DA) between the WT + HFD (*n* = 8) and KO + HFD (*n* = 8) groups. (B) The hierarchical clustering heat map showed that the difference in metabolites between the WT + HFD and KO + HFD groups (VIP > 1, *p*‐value < .05). 5‐diethoxyphosphoryl‐5‐methyl‐1‐pyrroline‐n‐oxide‐biotin was abbreviated as 5‐d‐5‐methyl‐1‐pyrroline‐n‐oxide‐biotin and 9‐deoxy‐9‐methylene‐16,16‐dimethylprostaglandin e2 was abbreviated as 9‐de‐dimethylprostaglandin e2. (C) Bubble diagram of KEGG pathway enrichment analysis between the WT + HFD and KO + HFD groups. (D) Hierarchical clustering heat map related to arachidonic acid metabolism in the WT + HFD and KO + HFD groups.

The metabolites of intestinal microbiota play a crucial role in mediating intestinal‐liver communication, we further analysed the intestinal microbiota‐related metabolites to explore the potential metabolites for KO‐regulated function. We applied metabolomics and analysed the intestinal contents of HFD‐treated WT and KO mice. A significant number of metabolites were found to differ between the two groups in both positive ion mode (Figures S5A and S6) and negative ion mode (Figures S5B and S7). The hierarchical clustering heat map in positive ion mode is shown in Figure S8. After establishing the relationship model between the expression of metabolites and sample categories by partial least square discrimination analysis (PLS‐DA), we found that the samples in the HFD and KO + HFD groups were significantly separated, confirming differences between them (Figure 4A). We then performed a hierarchical cluster analysis of significantly different metabolites (VIP > 1, *p*‐value < .05), and the metabolites clustered in the same cluster could share similar functions or be involved in the same metabolic process/pathway. The specific metabolites were displayed by the hierarchical clustering heat map, as shown in Figure 4B. To identify key pathways with notable impact, the KEGG pathway enrichment analysis was performed. Among the nine pathways with significant differences, ‘Arachidonic acid metabolism’ stood out as the most significantly altered pathway (Figure 4C). In addition to arachidonic acid metabolism, there are other pathways, such as ‘Protein digestion and absorption’, ‘Central carbon metabolism in cancer’, ‘Valine, leucine and isoleucine degradation/biosynthesis’, which may also play an important role in the normal function of the body. Further exploration of the differential metabolite associated with this pathway highlighted 3 upregulated metabolites (thromboxane b2, 11‐dehydrothromboxane b2, 5s‐14r‐lipoxin b4) and 4 downregulated ones (12‐oxo‐5z,8z,10e,14z‐eicosatetraenoic acid, arachidonic acid, leukotriene e4 and leukotriene f4) (Figure 4D).

### 3.4 MiR‐30a‐5p‐deficient and high‐fat‐fed mice accelerates hepatic metabolic disorders through COX and LOX pathways of arachidonic acid

**FIGURE 5:**
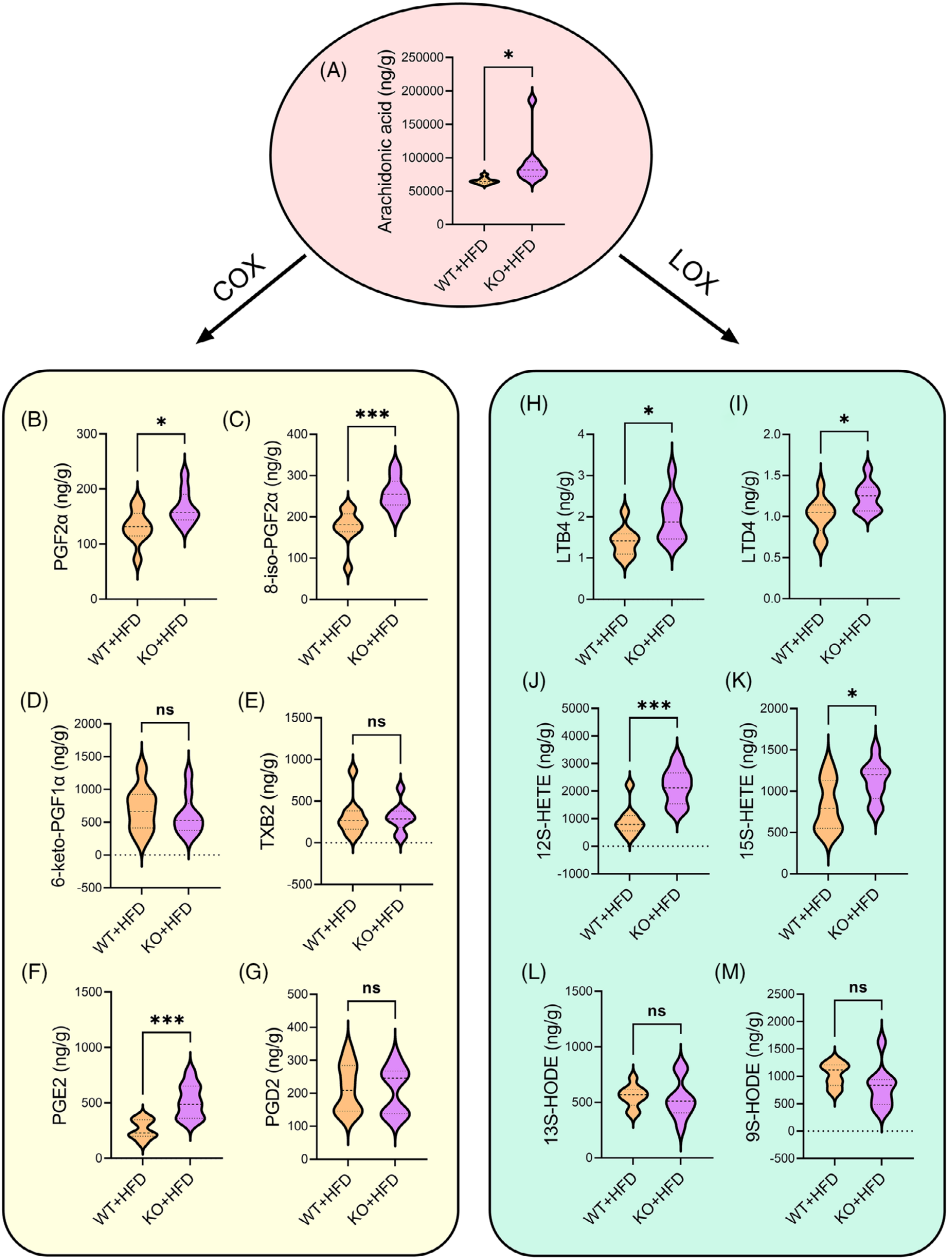
Targeted metabolomics of the liver tissues of HFD‐treated WT and KO mice. The targeted metabolomics results of arachidonic acid (A), and six substances of the COX pathway, including prostaglandin F2a (PGF2a) (B), 8‐iso‐prostaglandin F2a (8‐iso‐PGF2a) (C), 6‐keto‐prostaglandin F1a (6‐keto‐PGF1a) (D), thromboxane B2 (TXB2) (E), prostaglandin E2 (PGE2) (F) and prostaglandin D2 (PGD2) (G) in the WT + HFD group and the KO + HFD group (*n* = 9 in both groups). Six substances levels of the LOX pathway including leukotriene B4 (LTB4) (H), leukotriene D4 (LTD4) (I), 12s‐hydroxy eicosatetraenoic acid (12S‐HETE) (J), 15s‐hydroxy eicosatetraenoic acid (15S‐HETE) (K), 13s‐hydroxy octadecadienoic acid (13S‐HODE) (L) and 9s‐hydroxy octadecadienoic acid (9S‐HODE) (M) in the WT + HFD group and the KO + HFD group (*n* = 9 in both groups). *t*‐Test was used for two groups comparisons, NS showed no significant difference, ^*^ *p *< .05, ^**^ *p *< .01, ^***^ *p *< .001.

Arachidonic acid, crucial for human growth and development, possesses various physiological functions such as lowering cholesterol, increasing vascular elasticity, inhibiting platelet aggregation, and reducing blood viscosity. The cyclooxygenase (COX) and lipoxygenase (LOX) pathways are critical in arachidonic acid metabolism. Employing targeted metabolomics, we precisely measured the levels of arachidonic acid, along with COX‐ and LOX‐related metabolites in liver tissues of HFD‐treated WT and KO mice. Our findings revealed that the arachidonic acid level in the KO + HFD group exceeded that in the WT + HFD group (Figure 5A). Regarding the COX pathway, levels of prostaglandin F2a (PGF2a, Figure 5B), 8‐iso‐prostaglandin F2a (8‐iso‐PGF2a, Figure 5C), and prostaglandin E2 (PGE2, Figure 5F) in the liver of KO + HFD mice were higher than those of WT + HFD mice, the level of 6‐keto‐prostaglandin F1a (6‐keto‐PGF1a, Figure 5D) thromboxane B2 (TXB2, Figure 5E), and prostaglandin D2 (PGD2, Figure 5G) was not affected. In the LOX pathway, the levels of leukotriene B4 (LTB4, Figure 5H), leukotriene D4 (LTDI, Figure 5I), 12s‐hydroxy eicosatetraenoic acid (12S‐HETE, Figure 5J), 15s‐hydroxy eicosatetraenoic acid (15S‐HETE, Figure 5K) in the liver in KO + HFD group were higher than those in HFD group. Nevertheless, no significant changes were observed in 13s‐hydroxy octadecadienoic acid (13S‐HODE, Figure 5L) and 9s‐hydroxy octadecadienoic acid (9S‐HODE, Figure 5M). The above results demonstrated that KO mice, after HFD treatment, activated the LOX and COX pathways of arachidonic acid metabolism.

**FIGURE 6:**
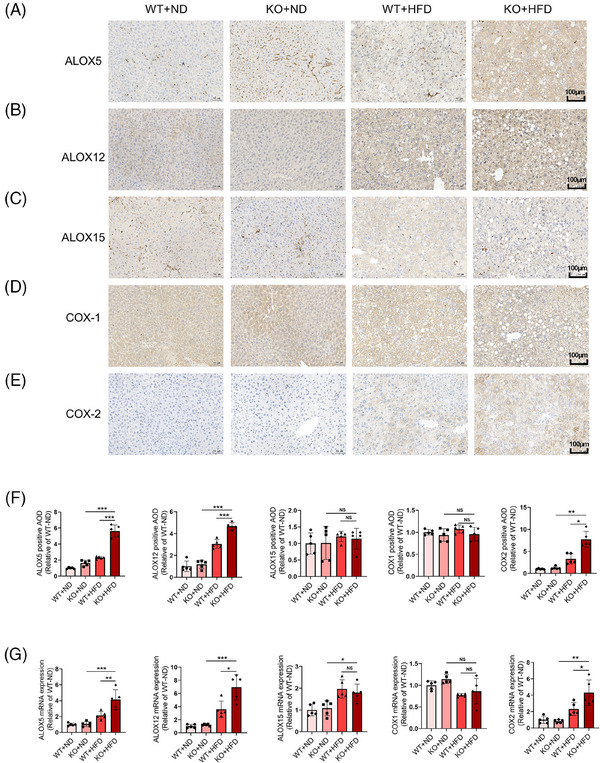
The expression level of proteins involved in the arachidonic acid metabolic pathway in liver tissues. Immunohistochemistry staining images of the expression levels of ALOX5 (A), ALOX12 (B), ALOX15 (C), COX‐1 (D), and COX‐2 (E) in liver tissues of WT or KO mice treatment with ND or HFD (scale bar: 100 µm). (F) The quantitative results of immunohistochemistry are determined by statistical average optical density (AOD). (G) The mRNA levels of ALOX5, ALOX12, ALOX15, COX‐1, and COX‐2 in liver tissue of WT or KO mice treated with ND or HFD were detected by real‐time PCR. Data are expressed as mean ± SD and one‐way ANOVA with Tukey's test was used for multiple comparisons, NS showed no significant difference, ^*^ *p *< .05, ^**^ *p *< .01, ^***^ *p *< .001.

According to the results of targeted metabolomics, LOX/COX pathways were considered as an important research direction. In LOX/COX pathways, some enzymes, including ALOX5, ALOX12, ALOX15, COX1 and COX2, affect the metabolic process of many factors and play a key role in the process of arachidonic acid metabolism. To further verify these findings, we detected the expression of key enzymes (ALOX5, ALOX12, ALOX15, COX1 and COX2) in liver tissues of WT/KO mice with ND/HFD‐fed. Immunohistochemistry staining indicated that in both the KO group and the WT + HFD group, the expression of ALOX5 (Figure 6A and F), ALOX12 (Figure 6B and F), and COX2 (Figure 6E and F) in liver tissues was significantly increased, with an even greater increase in the KO + HFD group. However, the expression of ALOX15 (Figure 6C and F) and COX1 (Figure 6D and F) showed no significant changes. Furthermore, we assessed the transcriptional levels by quantifying mRNA expression through qPCR, which exhibited similar patterns as observed at the protein level (Figure 6G). Based on the above results, we make a summary that miR‐30a‐5p KO mice, after HFD treatment, experienced accelerated hepatic metabolic disorder due to the COX/LOX‐based arachidonic acid metabolic pathway, and this process was closely related to the imbalance of intestinal microbial homeostasis.

### 3.5 Reintroducing miR‐30a‐5p reversed hepatic steatosis and arachidonic acid metabolism of KO mice after HFD

**FIGURE 7:**
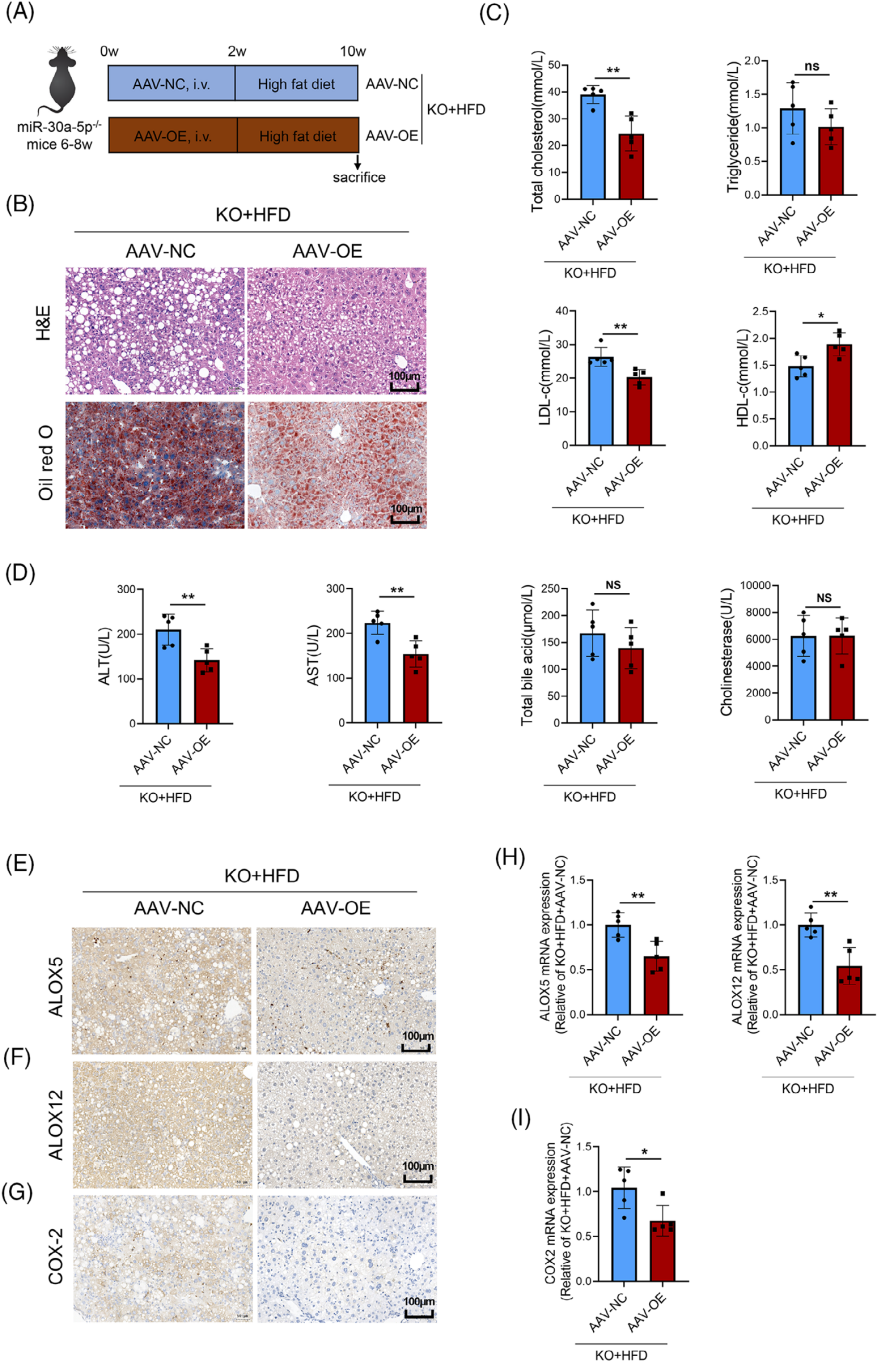
AAV‐OE reversed the HFD‐KO mice induced hepatic steatosis and COX/LOX pathways. (A) We injected 100 µL/each AAV‐NC or AAV‐OE into miR‐30a‐5p^−/−^ mice through the tail vein. Two weeks after injection, the mice began HFD for 8 weeks. (B) Representative images of H&E staining and Oil Red O of liver tissues in KO + HFD mice treatment with AAV‐NC or AAV‐OE (*n* = 5, scale bar: 100 µm). (C) The levels of total cholesterol, triglyceride, HDL‐c, and LDL‐c in the serum of KO + HFD mice treatment with AAV‐NC or AAV‐OE (*n* = 5). (D) The levels of ALT, AST, total bile acid, and cholinesterase in the serum of KO + HFD mice treatment with AAV‐NC or AAV‐OE (*n* = 5). (E) ALOX5 (F), ALOX12 and (G) COX‐2 in liver tissues of KO + HFD mice treatment with AAV‐NC or AAV‐OE (*n* = 5, scale bar: 100 µm). (H‐I) The mRNA levels of ALOX5, ALOX12, and COX‐2 in liver tissue of KO + HFD mice treatment with AAV‐NC or AAV‐OE (*n* = 5). Data are expressed as mean ± SD and one‐way ANOVA with Tukey's test was used for multiple comparisons, NS showed no significant difference, ^*^ *p *< .05, ^**^ *p *< .01.

To further investigate the role of miR‐30a‐5p on liver function and arachidonic acid metabolism, KO mice were injected AAV‐NC or AAV‐OE through the tail vein, and the expression of miR‐30a‐5p in mouse liver increased after AAV‐OE treatment (Figure S9). Two weeks later, these mice were HFD‐treatment for 8 weeks (Figure 7A). The results of HE and Oil Red O staining in mouse liver showed that AAV‐OE could reverse the hepatic steatosis caused by deletion of miR‐30a‐5p (Figure 7B). The levels of total cholesterol, triglyceride, LDL‐c, and HDL‐c were also proved the results (Figure 7C). Additionally, serum levels of ALT and AST decreased after AAV‐OE‐treatment, total bile acids and cholinesterase did not show significant changes (Figure 7D). Above results showed that reintroducing miR‐30a‐5p reversed significantly hepatic steatosis from HFD‐treated KO mice. The results of immunohistochemistry directly proved that the expression of ALOX5, ALOX12, and COX2 decreased significantly after restoring the expression of miR‐30a‐5p (Figure 7E–G). The expression of ALOX5, ALOX12 (Figure 7H), and COX2 (Figure 7I) at the transcription level also proved this conclusion. Therefore, reintroducing miR‐30a‐5p could reverse hepatic steatosis and arachidonic acid metabolism disorder caused by HFD and miR‐30a‐5p deletion.

## 4 DISCUSSION

NAFLD is considered as a significant global health concern, with potential progression to cirrhosis, liver cancers, and concurrent cardiovascular diseases, ultimately leading to mortality.^18^ The pathogenesis of NAFLD is no longer limited to fat accumulation in the liver, and its complexity has been gradually identified.^19^ In this study, our findings demonstrate several key points: (1) miR‐30a‐5p deletion aggravated hepatic steatosis and lipid disorder induced by an HFD in mice; (2) gut microbiota participated in the regulation of hepatic steatosis in the context of miR‐30a‐5p; (3) gut microbiota metabolism‐related arachidonic acid metabolic pathway contributed to miR‐30a‐5p‐regulated hepatic steatosis and lipid disorder; (4) reintroducing miR‐30a‐5p reversed hepatic steatosis and arachidonic acid metabolism disorder caused by HFD and miR‐30a‐5p deletion.

MicroRNAs (miRNAs) regulate the function of different cells and are utilised as biomarkers for diagnosing and predicting the prognosis of many diseases.^20^, ^21^ MiR‐30a‐5p has been proved widely involved in the pathogenesis of tumours, cardiovascular diseases, and kidney disorders.^10^, ^22^, ^23^ Specifically, a liver‐related study found that miR‐30a‐5p improved the drug resistance of liver tumours to sorafenib by targeting CLCF1.^24^ Our previous study demonstrated that over‐expression of miR‐30a‐5p mitigated atherosclerosis and improved lipid metabolism in ApoE^−/−^ mice.^15^ However, the role of miR‐30a‐5p on liver function remains unclear. In this study, we constructed miR‐30a‐5p knockout (KO) mice and found KO had more fat accumulation in liver tissue and higher circulating cholesterol and LDL levels than WT mice.

Studies have shown that intestinal barrier damage in patients with NAFLD led to changes in intestinal bacteria, accelerated hepatic inflammation and the progress of NAFLD, indicating the importance of the gut‐liver axis.^4^, ^25^ In this study, we observed significant differences in the intestinal microorganisms between miR‐30a‐5p‐deficient mice and WT mice after exposure to a high‐fat diet, which may be an important reason for the difference in liver phenotype between the two groups. Our intestinal microbiota transplantation experiments confirmed that intestinal microbes from miR‐30a‐5p‐deficient mice could alter liver structure and function. The gut‐liver axis involves two‐way interactions: damage to the intestine allows bacteria and their metabolites to enter the portal vein, leading to liver damage, while the liver can impact the intestinal function through secreting bile acids and other active substances.^26^, ^27^, ^28^ Sequencing results proved that the change of *Akkermansia muciniphila* was the most significant among WT/KO mice fed with HFD. It was previously reported that *Akkermansia muciniphila* has proven to contribute to alleviate hepatic steatosis, obesity, type 2 and type 1 diabetes mellitus, intestinal inflammation and different cancers (colon cancer, response to immune checkpoints) in mice.^16^, ^29^ Because inflammation is the root cause of many of above diseases, *Akkermansia muciniphila* regulated immunity by maintaining a healthy intestinal barrier, thus inhibiting the activation of inflammation.^30^ These may partly explain the sensitivity of *Akkermansia muciniphila* in KO‐HFD mice and the regulatory effect of miR‐30a‐5p on intestinal bacteria, but the specific mechanism research needs more experimental design to prove. Here, we draw a preliminary conclusion that miR‐30a‐5p may play a key role in mediating the relationship between the liver and the intestine.

Changes in the intestinal microbial community can cause changes in its metabolites, and some signals may also be transmitted through these metabolites.^25^, ^28^ In this study, we analysed the different intestinal metabolites in mice and found that the arachidonic acid pathway may be involved in the regulation of NAFLD by miR‐30a‐5p. Differential metabolites related to arachidonic acid signalling pathway included thromboxane b2, 11−dehydrothromboxane b2, 5(s),14(r)−lipoxin b4, 12−oxo−5z,8z,10e,14z−eicosatetraenoic acid, arachidonic acid (peroxide free), leukotriene (LT) e4, LTf4. Arachidonic acid, a common and abundant polyunsaturated fatty acid in mammalian cell membranes,^31^ is a precursor to a variety of bioactive carotenoids (such as prostaglandins, thromboxane A2, and leukotriene), and it plays an important role in immune function, inflammatory responses, and allergy reactions.^32^ Studies have shown that arachidonic acid metabolites are involved in the process of hepatic steatosis, with the pro‐inflammatory LOX pathway acting as a major driver of NAFLD progression.^33^ Arachidonic acid is released from cell membranes by phospholipase A2 (PLA2) and is mainly metabolised through COX, LOX and cytochrome P450 (CYP) pathways.^34^ COX‐1/2 converts arachidonic acid into PGG2 and PGH2, which are further metabolised into various prostaglandins, including PGD2, PGE 2α and PGF2α through corresponding synthases.^35^ LOX (including 5‐LOX, 12‐LOX and 15‐LOX) catalyses the deoxygenation of arachidonic acid to produce hydroperoxyl eicosatetraenoic acids (HPETEs), which transform into hydroxy eicosatetraenoic acids (HETEs), LTs and lipoxins (LXs).^36^ The CYP pathway mainly promotes the metabolism of lipophilic xenobiotics, yielding HETEs and epoxeicosatrienoic acids (EETs).^37^ We also detected the level of arachidonic acid metabolites by targeted metabolomics and found that miR‐30a‐5p deletion increased the levels of arachidonic acid and specific COX and LOX pathway metabolites. COX pathway metabolites with elevated levels included PGF2α, 8‐iso‐PGF2α, and PGE2, while LOX pathway metabolites with elevated levels included LTB4, LTD4, 12S‐HETE and 15S‐HETE.

The diversity of arachidonic acid metabolites is governed by specific metabolic enzymes. We assessed the expression levels of COX and LOX‐related enzymes to understand their impact. Following miR‐30a‐5p deletion and HFD induction, we observed significant alterations in the expression of ALOX5, ALOX12 and COX2, while the expression of ALOX15 and COX1 remained unaffected. LOXs catalyse the production of lipid peroxide. ALOX5 converts arachidonic acid into 5‐HETE, LTA4, LTB4 and LTC4,^38^ whereas ALOX12 transforms free arachidonic acid into 12‐HETE.^39^ Tak et al. proved that the weakened effect of miR‐15a on ALOX12 led to liver lipid peroxidation and ferroptosis, which further aggravated acute liver damage.^40^ Zhong et al. also found that the increase of ALOX12 aggravated mitochondrial lipid peroxidation during liver ischemia‐reperfusion injury.^41^ Similarly, our findings revealed that the deletion of miR‐30a‐5p after HFD increased the expression of ALOX5 and ALOX12, which further led to hepatic steatosis through lipid peroxidation of the liver. On the other hand, COX is responsible for the biosynthesis of prostaglandin from arachidonic acid.^42^ COX1, a constituent of the COX enzyme family, typically exhibits a protective role against liver injury.^43^, ^44^ COX2 can be induced by various stimuli such as cytokines, growth factors, hormones and tumour promoters.^45^ Conversely, COX2 is highly expressed in hepatocellular carcinoma, with its inhibition showing promise as a therapeutic approach.^46^, ^47^ In our study, the liver damage caused by the deletion of miR‐30a‐5p following HFD was associated with increased expression of ALOX5, ALOX12, and COX2, which could involve in the lipid peroxidation and the activation of inflammatory reaction in the liver (Figure S10).

In summary, we found that miR‐30a‐5p^−/−^ mice displayed more severe hepatic steatosis and dyslipidaemia compared to WT mice after an HFD. Changes in intestinal microbiota are involved in miR‐30a‐5p's role in liver injury in mice. Through analysing intestinal metabolites, we found a link between the arachidonic acid pathway and miR‐30a‐5p. Our results also showed that miR‐30a‐5p regulated gut microbiota through ALOX5, ALOX12 and COX2 – key enzymes of the arachidonic acid pathway – affecting hepatic steatosis and dyslipidaemia in mice. Importantly, reintroducing miR‐30a‐5p reversed hepatic steatosis and COX and LOX pathways of arachidonic acid.

## AUTHOR CONTRIBUTIONS

Ruiying Wang, Yan Wang and Gang Li designed the study. Ruiying Wang and Xiaocheng Zhang completed the experimental process and data collection. Ruiying Wang and Gang Li wrote and edited the manuscript. Yutian Wang, Yijun Lin and Yuling Zhou were responsible for statistical analyses. All authors reviewed and approved the final manuscript.

## CONFLICT OF INTEREST STATEMENT

The authors declare they have no conflicts of interest.

## ETHICS STATEMENT

All experiments of animals in this study were approved by the Ethics Committee of the Laboratory Animal Management and Ethics Committee of Xiamen University (Approval NO. XMULAC20190120).

## Supporting information



Supporting Information

## Data Availability

The data supporting the findings of this study are available within the article and its supplementary information files. All original data for this study can be obtained from the corresponding author.
