# Supplementary material for: Gut microbiota of miR‐30a‐5p‐deleted mice aggravate high‐fat diet‐induced hepatic steatosis by regulating arachidonic acid metabolic pathway
Source: Clin Transl Med. 2024 Oct 3;14(10):e70035. doi: 10.1002/ctm2.70035 (PMC11447637; doi:10.1002/ctm2.70035)
Supplement: Supplementary file 1 — Supporting Information [file CTM2-14-e70035-s001.docx]

**Gut microbiotas of miR-30a-5p-deleted mice aggravate** **high-fat diet-induced hepatic** **steatosis by regulating the arachidonic acid metabolic pathway**

Ruiying Wang^1,2*^, Xiaocheng Zhang^1^, Yutian Wang^3^, Yijun Lin^1,2^, Yuling Zhou^1,2^, Yan Wang^1,2*^, Gang Li^1,2*^

1. Xiamen Cardiovascular Hospital of Xiamen University, School of Medicine, Xiamen University, Xiamen, Fujian, People's Republic of China.

2. Xiamen Key Laboratory of Cardiovascular Diseases, Xiamen, Fujian, People's Republic of China.

3. Department of Cardiology, Nanfang Hospital, Southern Medical University, Guangzhou, Guangdong, People's Republic of China.

**Running title:** Roles of miR-30a-5p on hepatic steatosis

***Correspondence to:**

Ruiying Wang, Xiamen Cardiovascular Hospital of Xiamen University, School of Medicine, Xiamen University, Xiamen, Fujian (361000), China. Email address: wangruiying@xmu.edu.cn.

Yan Wang, Xiamen Cardiovascular Hospital of Xiamen University, School of Medicine, Xiamen University, Xiamen, Fujian (361000), China. Email address: wy@medmail.com.cn.

Gang Li, Xiamen Cardiovascular Hospital of Xiamen University, School of Medicine, Xiamen University, Xiamen, Fujian (361000), China. Email address: ligang@xmu.edu.cn.

**Supplementary Figures**


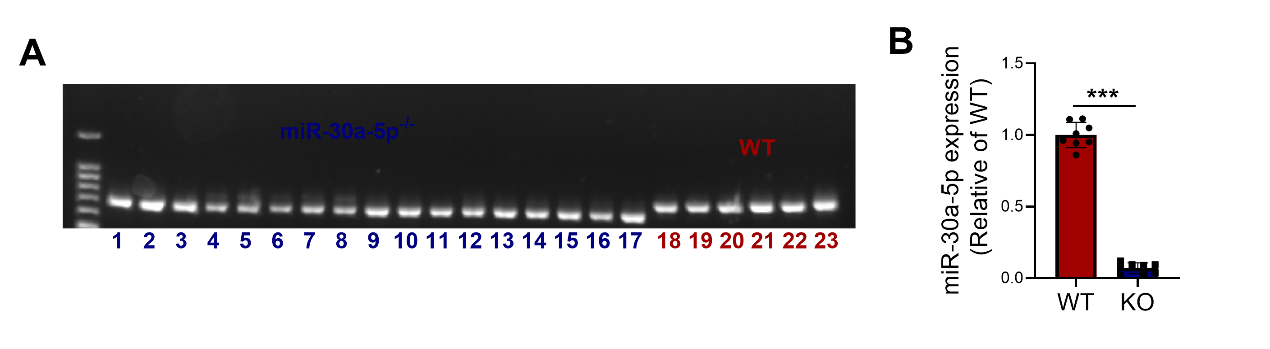


**Supplementary figure 1.** Identification of miR-30a-5p expression in miR-30a-5p^-/-^ mice. **(A)** genotyping of miR-30a-5p knockout (miR-30a-5p^-/-^) mice. Bands 1-16 indicated miR-30a-5p^-/-^ mice and bands 17-23 indicated the wild-type (WT) mice. **(B)** qRT-PCR quantified the miR-30a-5p expression in the liver of WT and KO mice. Data are expressed as mean ± SD (n = 8) and ﻿Student’s t-test was used for statistical analysis, ^***^*P*<0.001.


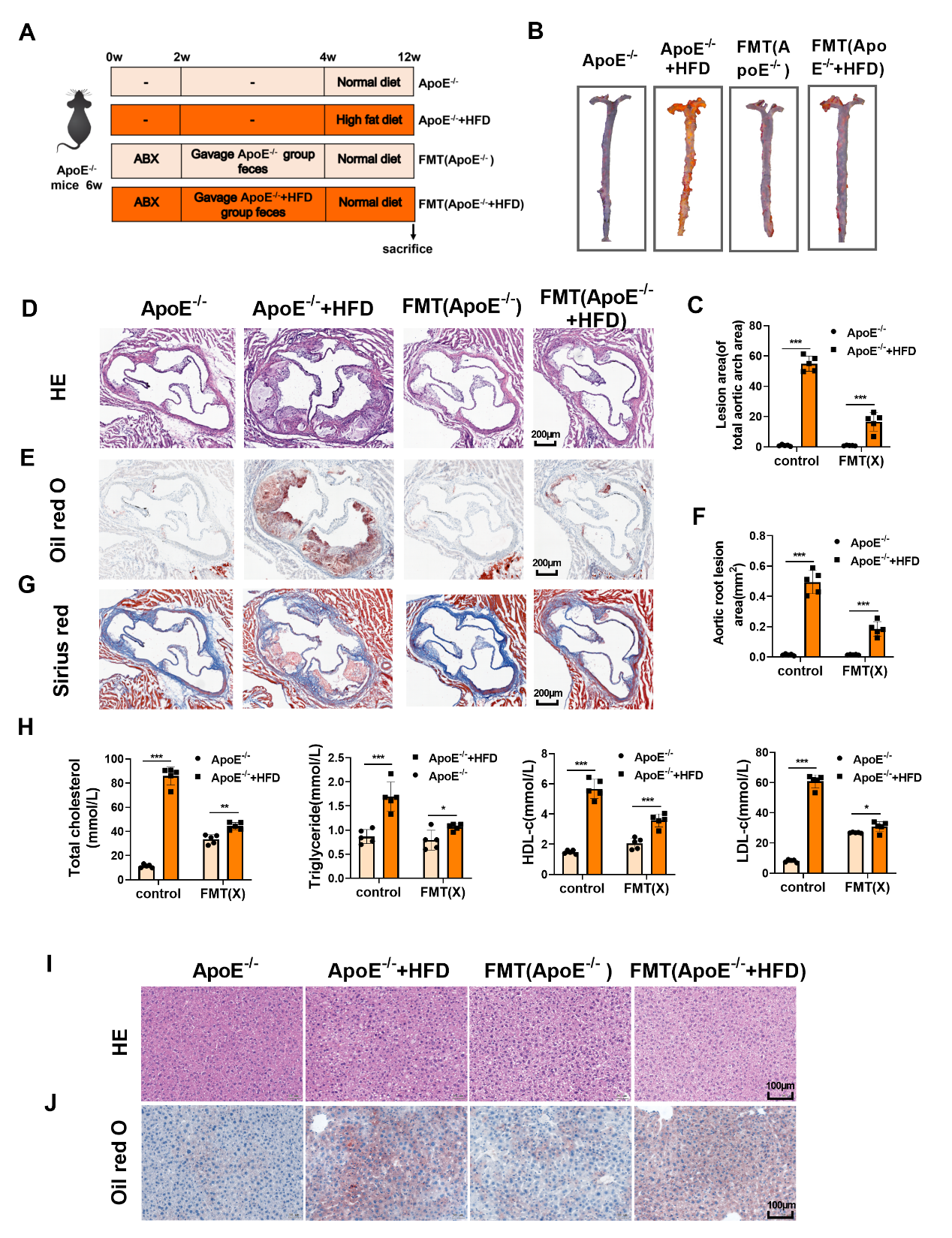


**Supplementary figure 2. ApoE^-/-^ mice transplanted with intestinal microorganisms in HFD group can induce plaque and dyslipidemia.** (A) ApoE^-/-^ mice were fed with high fat or normal diet for 2 months to complete the model. In addition, other ApoE^-/-^ mice were cleared of intestinal microorganisms with ABX for 2 weeks, and continued to be fed with normal diet for 2 months after transplanting intestinal microorganisms in control group or HFD group for 2 weeks. (B) Representative images and (C) analysis of plaque in the aortic arch of ApoE^-/-^, ApoE^-/-^+HFD, FMT(ApoE^-/-^) and FMT(ApoE^-/-^+HFD) group (n=5). (D) Representative images of HE staining of aortic root in ApoE^-/-^, ApoE^-/-^+HFD, FMT(ApoE^-/-^) and FMT(ApoE^-/-^+HFD) group (n=5, Scale bar: 200 μm). (E) Representative images and (F) analysis of oil red O staining of aortic root in ApoE^-/-^, ApoE^-/-^+HFD, FMT(ApoE^-/-^) and FMT(ApoE^-/-^+HFD) group (n=5, Scale bar: 200 μm). (G) Representative images of masson staining of aortic root in ApoE^-/-^, ApoE^-/-^+HFD, FMT(ApoE^-/-^) and FMT(ApoE^-/-^+HFD) group (n=5, Scale bar: 200 μm). (H) The levels of total cholesterol, triglyceride, HDL-c and LDL-c in serum of ApoE^-/-^, ApoE^-/-^+HFD, FMT(ApoE^-/-^) and FMT(ApoE^-/-^+HFD) group (n=5). (I) Representative images of HE staining and (J) oil red O of liver tissue in ApoE^-/-^, ApoE^-/-^+HFD, FMT(ApoE^-/-^) and FMT(ApoE^-/-^+HFD) group (n=5, Scale bar: 100 μm). Data are expressed as mean ± SD and ﻿one-way ANOVA with Tukey’s test was used for multiple comparisons, NS showed no significant difference, ^*^*P*<0.05, ^**^*P*<0.01, ^***^*P*<0.001.


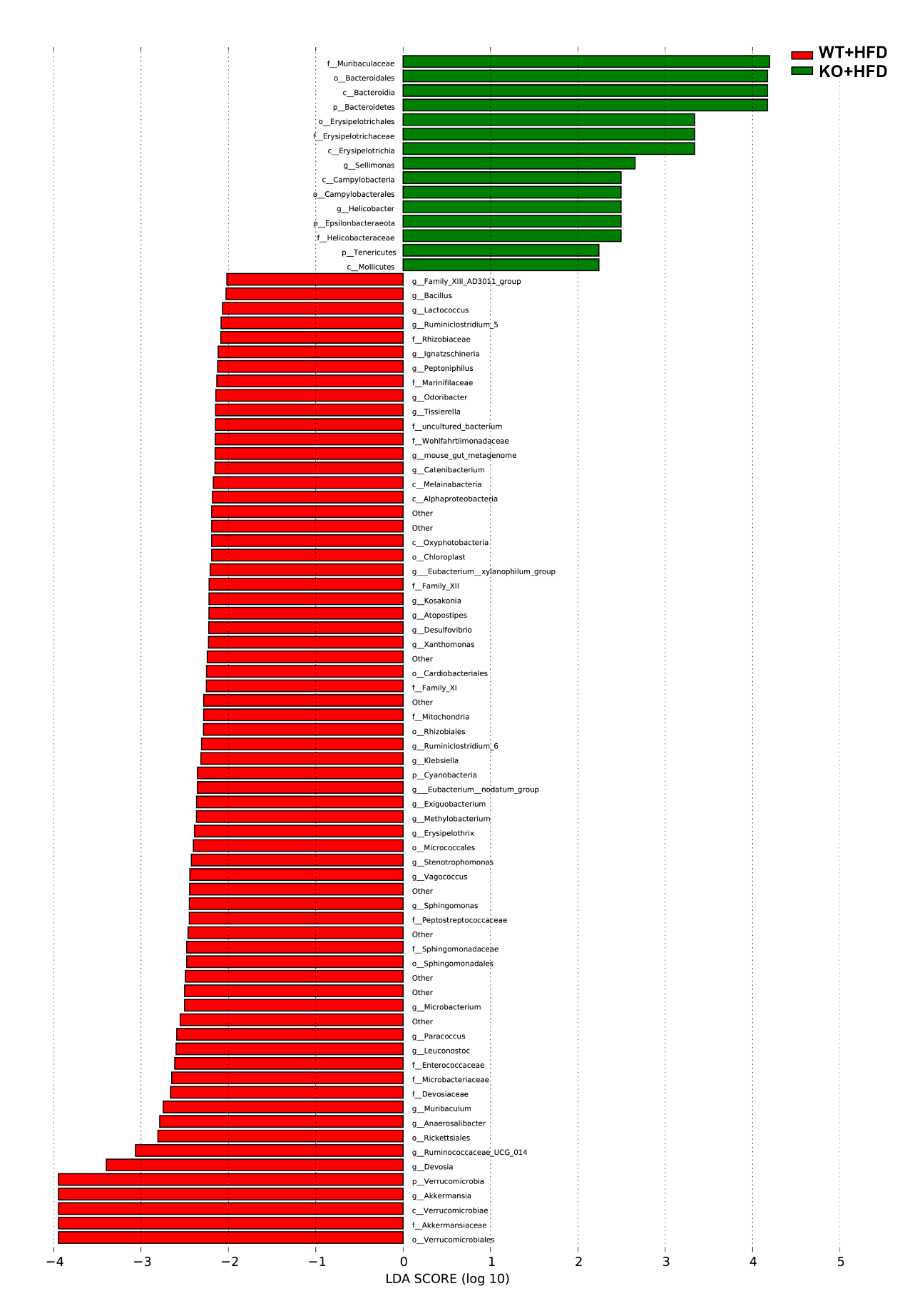


**Supplementary figure 3.**  **LDA Effect Size analysis** **of the** **16s rDNA sequence of the fecal microbiotas in the HFD-treated WT and KO mice showed the species that differ significantly between the** **two groups.** The bar graph length represents the LDA value, with larger LDAs indicating more significant differences. Species with LDA greater than 2 were biomarkers with statistical differences between groups.


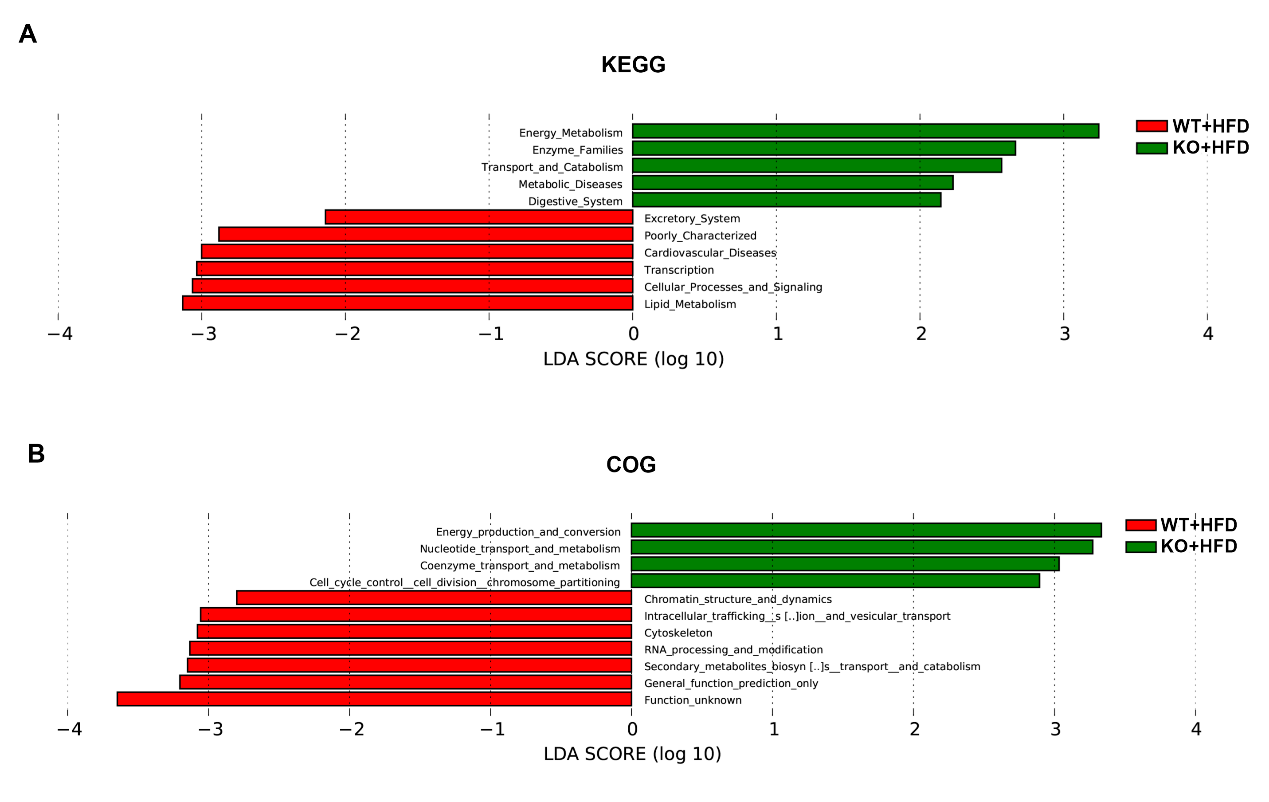


**Supplementary figure 4.** **Function prediction of the 16s rDNA sequence of the fecal microbiotas** **in the WT+HFD and KO+HFD groups. (A)** The results of LDA Effect Size (LEfSe) analysis based on KEGG function prediction were shown including the histogram of LDA value distribution and the comparison chart of functional items with statistical differences between the WT+HFD and KO + HFD groups. **(B)** The results of LEfSe analysis based on COG function prediction were shown including the histogram of LDA value distribution and the comparative graph of functional items with statistical differences between the WT + HFD and KO+HFD groups.


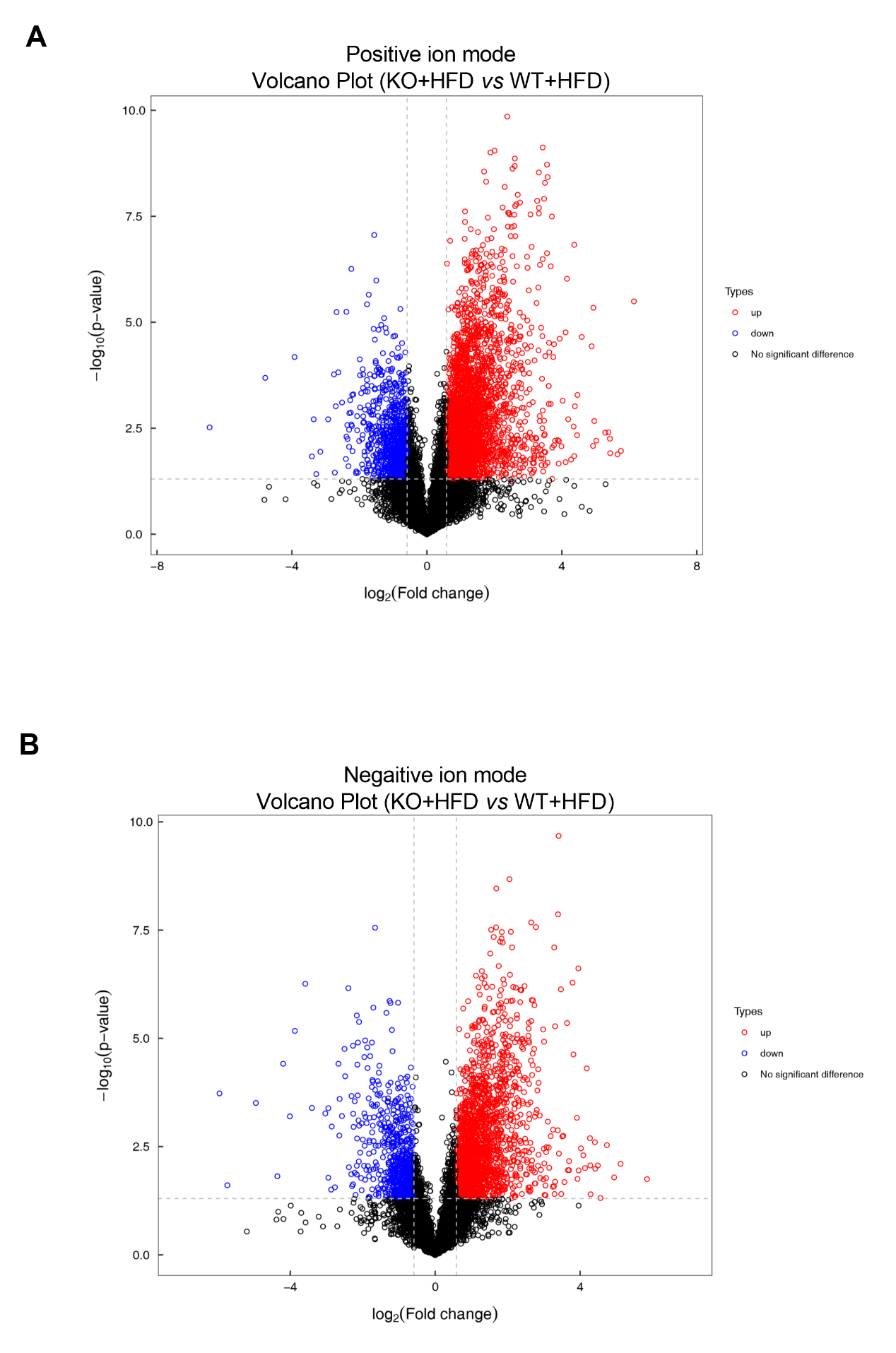


**Supplementary figure 5. The volcano plot showed the difference in metabolites between the WT + HFD and KO + HFD groups.** Based on univariate analysis, all metabolites (including unidentified metabolites) detected in the positive **(A)** and negative **(B)** ion modes were analyzed. The differential metabolites with FC >1.5 or FC < 0.67 and p-value < 0.05 were displayed in the volcano plot. Up-regulated significantly different metabolites were shown in red, down-regulated significantly different metabolites were shown in blue, and non-significantly different metabolites were shown in black.


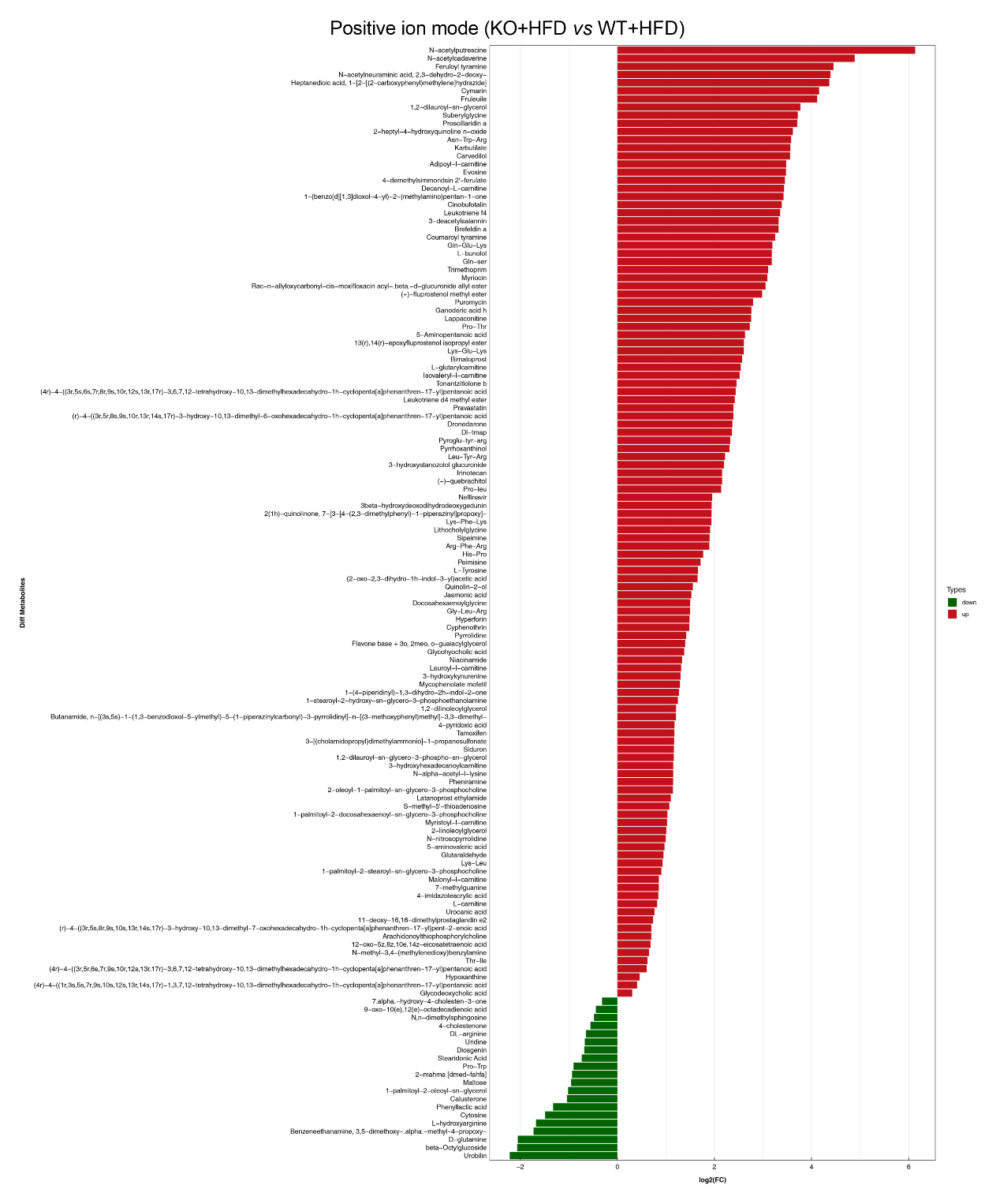


**Supplementary figure 6.** Bar graph of differential multiples of significant differential metabolites in positive ion mode. The metabolites of significant difference (in accordance with the screening criteria of OPLS-DA VIP > 1 and p-value < 0.05) were displayed by bar graph. Red indicated up-regulation of differential metabolites and green indicated down-regulation of differential metabolites.


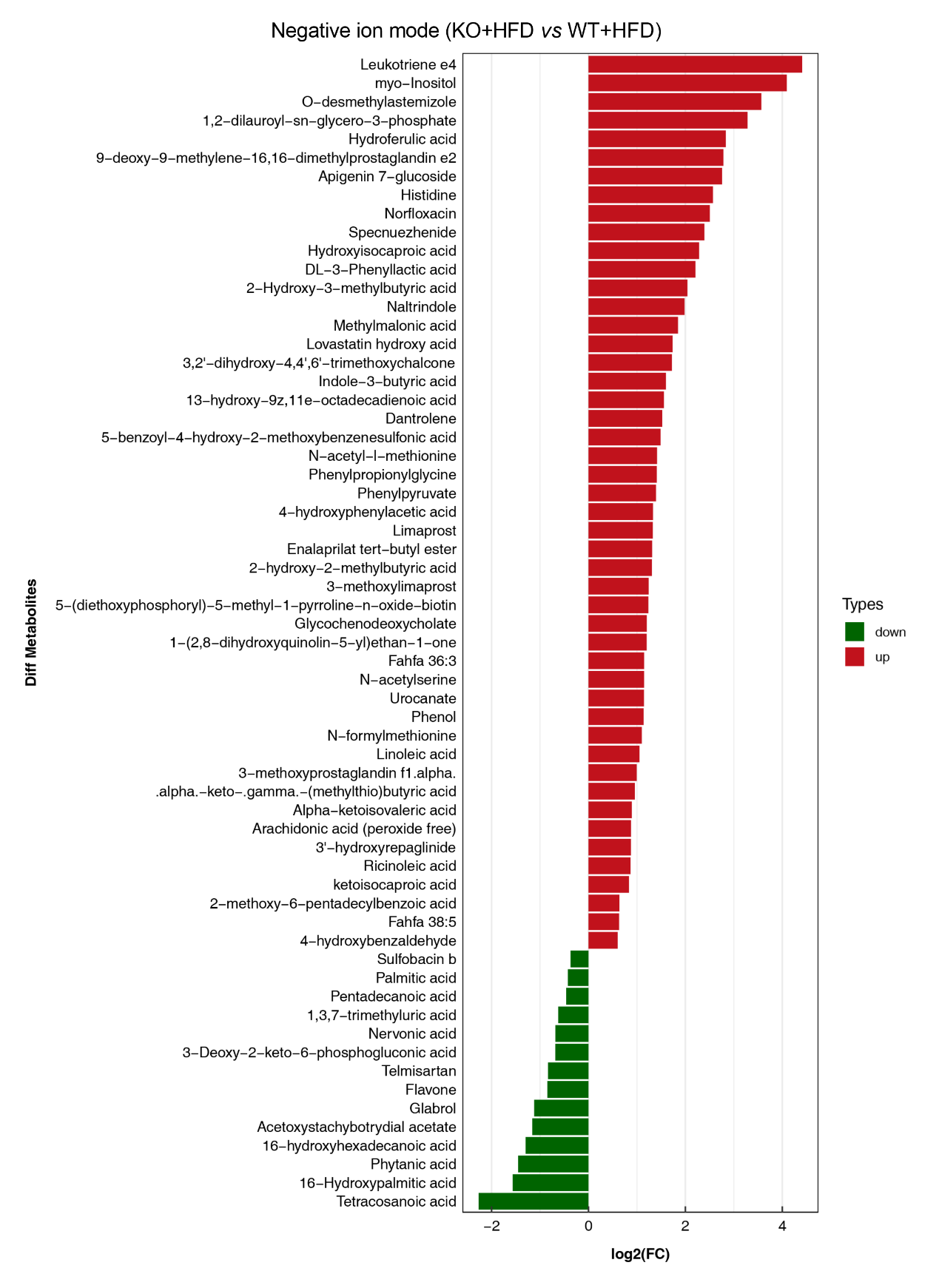


**Supplementary figure 7.** **Bar graph of differential multiples of significant differential metabolites between the WT + HFD and KO + HFD groups in negative ion mode**. The metabolites of significant difference (in accordance with the screening criteria of OPLS-DA VIP > 1 and p-value < 0.05) were displayed by bar graph. Red indicated up-regulation of differential metabolites and green indicated down-regulation of differential metabolites.


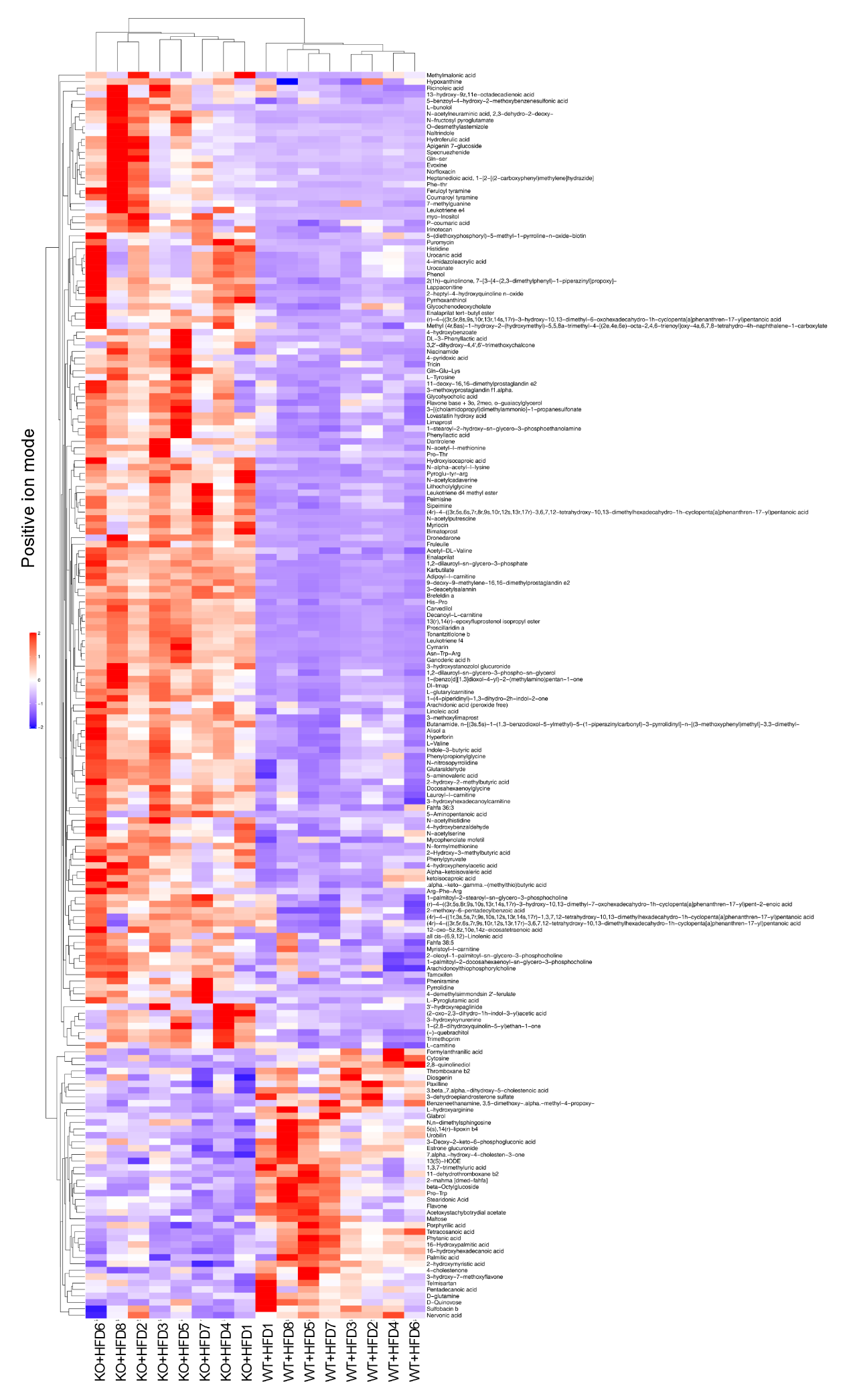


**Supplementary figure 8.** **The hierarchical clustering heat map in positive ion mode showed** **the different metabolites of the fecal** **microbiotas between the WT + HFD and KO + HFD groups.** The difference in metabolites between HFD and KO + HFD groups was consistent with VIP > 1, p-value < 0.05 (n = 8).


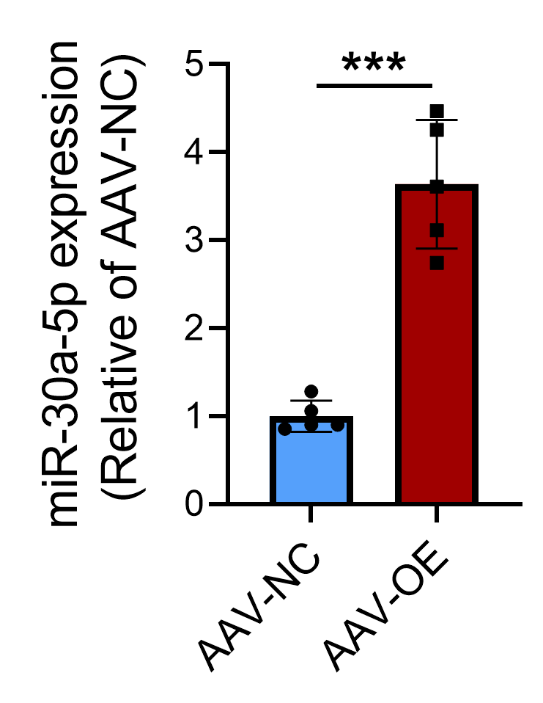


**Supplementary figure 9.** Identification of miR-30a-5p expression in KO mice after reintroducing miR-30a-5p. qRT-PCR quantified the miR-30a-5p expression in the liver of KO mice treatment with AAV-NC or AAV-OE. Data are expressed as mean ± SD (n = 5) and ﻿Student’s t-test was used for statistical analysis, ^***^*P*<0.001.


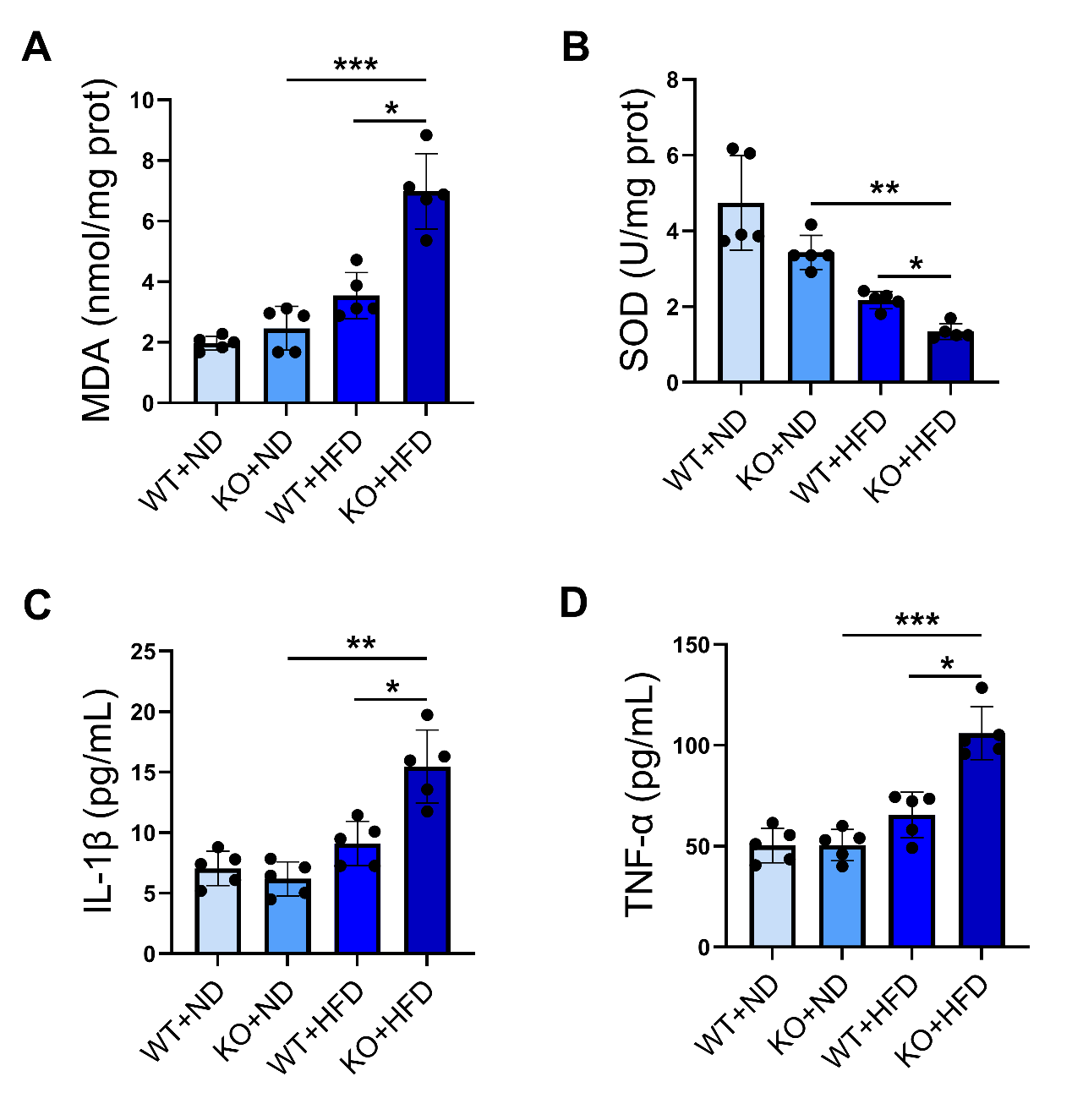


**Supplementary figure 10** The deletion of miR-30a-5p aggravated liver lipid peroxidation and inflammatory reaction after HFD. (A) MDA levels in liver tissues were detected in the WT/KO mice treated with ND or HFD (n = 5). (B) SOD levels in liver tissues were detected in the WT/KO mice treated with ND or HFD (n = 5). (C) IL-1β levels in liver tissues were detected in the WT/KO mice treated with ND or HFD (n = 5). (D) TNF-α levels in liver tissues were detected in the WT/KO mice treated with ND or HFD (n = 5). Data are expressed as mean ± SD and one-way ANOVA with Tukey’s test was used for multiple comparisons, NS indicated no significant difference, ^*^*P*<0.05, ^**^*P*<0.01, ^***^*P*<0.001.
